# The American Paddlefish Genome Provides Novel Insights into Chromosomal Evolution and Bone Mineralization in Early Vertebrates

**DOI:** 10.1093/molbev/msaa326

**Published:** 2020-12-17

**Authors:** Peilin Cheng, Yu Huang, Yunyun Lv, Hao Du, Zhiqiang Ruan, Chuangju Li, Huan Ye, Hui Zhang, Jinming Wu, Chengyou Wang, Rui Ruan, Yanping Li, Chao Bian, Xinxin You, Chengcheng Shi, Kai Han, Junming Xu, Qiong Shi, Qiwei Wei

**Affiliations:** 1 Key Laboratory of Freshwater Biodiversity Conservation, Ministry of Agriculture and Rural Affairs of P.R. China, Yangtze River Fisheries Research Institute, Chinese Academy of Fishery Sciences, Wuhan, China; 2 Shenzhen Key Lab of Marine Genomics, Guangdong Provincial Key Lab of Molecular Breeding in Marine Economic Animals, BGI Academy of Marine Sciences, BGI Marine, BGI, Shenzhen, China; 3 Key Laboratory of Sichuan Province for Fishes Conservation and Utilization in the Upper Reaches of the Yangtze River, Neijiang Normal University, Neijiang, China; 4 BGI-Qingdao, BGI-Shenzhen, Qingdao, China; 5 Laboratory of Marine Genomics, School of Life Sciences and Oceanography, Shenzhen University, Shenzhen, China

**Keywords:** American paddlefish, sturgeon and paddlefish, early vertebrates, whole-genome duplication, chromosome evolution, bone mineralization

## Abstract

Sturgeons and paddlefishes (Acipenseriformes) occupy the basal position of ray-finned fishes, although they have cartilaginous skeletons as in Chondrichthyes. This evolutionary status and their morphological specializations make them a research focus, but their complex genomes (polyploidy and the presence of microchromosomes) bring obstacles and challenges to molecular studies. Here, we generated the first high-quality genome assembly of the American paddlefish (*Polyodon spathula*) at a chromosome level. Comparative genomic analyses revealed a recent species-specific whole-genome duplication event, and extensive chromosomal changes, including head-to-head fusions of pairs of intact, large ancestral chromosomes within the paddlefish. We also provide an overview of the paddlefish *SCPP* (secretory calcium-binding phosphoprotein) repertoire that is responsible for tissue mineralization, demonstrating that the earliest flourishing of *SCPP* members occurred at least before the split between Acipenseriformes and teleosts. In summary, this genome assembly provides a genetic resource for understanding chromosomal evolution in polyploid nonteleost fishes and bone mineralization in early vertebrates.

## Introduction

Since the first fish genome of the fugu was released in 2002 (Aparicio et al. 2002), more than 60 fish genomes have been published (Ravi and Venkatesh 2018; Bian et al. 2019). The spotted gar (Braasch et al. 2016) and the sterlet (Cheng et al. 2019; Du et al. 2020) are the only nonteleost ray-finned fishes reported to date. Acipenseriformes (sturgeons and paddlefishes), as an important order of nonteleosts, is estimated to have originated from 300 to 350 Ma or even earlier (Hughes et al. 2018). There are only two extant paddlefish species, the Chinese paddlefish (*Psephurus gladius*, declared functionally extinct very recently; Mei et al. 2020; Zhang et al. 2020) and the American paddlefish (*Polyodon spathula*). Therefore, as perhaps the only living species within the family, the American paddlefish is valuable as a representative species for understanding early vertebrate evolution.

The evolution of vertebrate ancestors was accompanied by two rounds (1R and 2R) of whole-genome duplication (WGD; Dehal and Boore 2005). A third WGD (3R) occurred at 320 Ma was defined in teleosts (Vandepoele et al. 2004), which account for more than 99% of all ray fins (Actinopterygia), but not in the basal fishes including sturgeons and paddlefishes. However, Acipenseriformes is known to be the only lineage among the basal fishes with their own lineage-specific WGDs that happened more recently (Vandepoele et al. 2004; Crow et al. 2012). It is also believed that the WGDs that occurred in paddlefishes and in sturgeons are two independent events based on studies of *Hox* clusters and several other genes (Crow et al. 2012; Cheng et al. 2019). Therefore, more genomic studies are required to verify the existence and timing of the WGDs, and to interpret subsequent effects caused by such lineage-specific events.

One consequence of WGD is the increasing number of chromosomes. American paddlefish has a significantly higher chromosome number (2*n* = 120; Symonová et al. 2017) than other fishes (most with either 48 or 50 chromosomes; Mank and Avise 2006), which is an interesting common feature shared with Acipenseriformes species. Previous studies reported that paddlefish and sturgeon genomes contain many small dot-like chromosomes (defined as microchromosomes) that are significantly different from the relatively longer microchromosomes in birds and reptiles (Deakin and Ezaz 2019; O’Connor et al. 2019). However, there is no clear boundary between macro- and microchromosomes in paddlefishes and sturgeons, and the causes for such an interesting pattern are not well known, although many efforts have been made in previous karyotypic studies (Symonová et al. 2017).

Sturgeons and paddlefishes have been referred to as “living fossils” due to their conserved evolution and few morphological modifications (Liu et al. 2018). Although as ray-finned fishes, they present many morphological similarities with sharks in Chondrichthyes, especially the almost entirely cartilaginous bones (Davesne et al. 2020). The cause for such an ancient phenotype is unclear, but the cartilaginous nature of these fishes was thought to be a derived character since sturgeon ancestors have bony skeletons (Helfman et al. 2009). There is a hypothesis that the absence of secretory calcium-binding phosphoprotein (*SCPP*) gene is responsible for the absence of bone from the endoskeleton of cartilaginous fishes (Venkatesh et al. 2014). However, whether this hypothesis is applicable to the ray-finned paddlefish and sturgeons needs further investigation.

Nonetheless, paddlefish genome has remained largely unexplored due to its polyploidy and the presence of many microchromosomes, which hinders in-depth evolutionary and biological studies of this threatened and commercially valuable fish. Therefore, in the present study, we performed whole-genome sequencing to obtain a high-quality genome assembly of the American paddlefish at a chromosome level. With this genome and the results from comparative genomic analyses, we attempted to answer the following critical questions: 1) What is the chromosomal evolutionary pattern in paddlefish? 2) How were chromosomes rearranged after independent lineage-specific WGDs in paddlefish and sterlet in comparison to the spotted gar that experienced neither the TGD (teleost genome duplication; Bian et al. 2016) nor a species-specific WGD? 3) Do the previously reported bone mineralization-related *SCPP* genes exist in the American paddlefish and the sterlet?

## Results

### Summary of the Primary Genome Assembly and Annotation

We applied both short and long reads to generate the genome assembly of the American paddlefish. In total, our sequencing of 462.3-Gb raw data (supplementary table S1, Supplementary Material online) had a coverage of 300× over the 1.56-Gb estimated genome size (supplementary fig. S1, Supplementary Material online) based on a 17-mer analysis (Liu et al. 2013). After initial contig construction, long reads-based scaffolding, and additional scaffold connection, we obtained a final assembled genome of 1.54 Gb, accounting for 98.7% of the estimated size, with a contig N50 length of 4.30 Mb and a scaffold N50 of 4.86 Mb (supplementary table S2, Supplementary Material online).

Through GC distribution checking, we observed that the reads used for the genome assembly displayed a homogeneous GC distribution, indicating good quality without pollution (supplementary fig. S2, Supplementary Material online). In a BUSCO validation, total completeness of the primary genome assembly was estimated to be 93.7%, including 50.9% single-copy BUSCOs and 42.8% duplicates. The fragmented BUSCOs were estimated to be 2.3%, and the rest (4.0%) were missing BUSCOs (supplementary table S3, Supplementary Material online).

For the repeat annotation, a total of 38.4% of the assembled genome was annotated as repeat sequences (supplementary tables S4 and S5, Supplementary Material online). By integrating the three strategies (homology, de novo, and transcriptome based) of gene annotation, we predicted 26,017 protein-encoding genes (supplementary table S6, Supplementary Material online), of which 99.50% were annotated with at least one functional term from the searched biological databases (supplementary table S7, Supplementary Material online).

### Chromosome-Level Genome Assembly

**Fig. 1.**
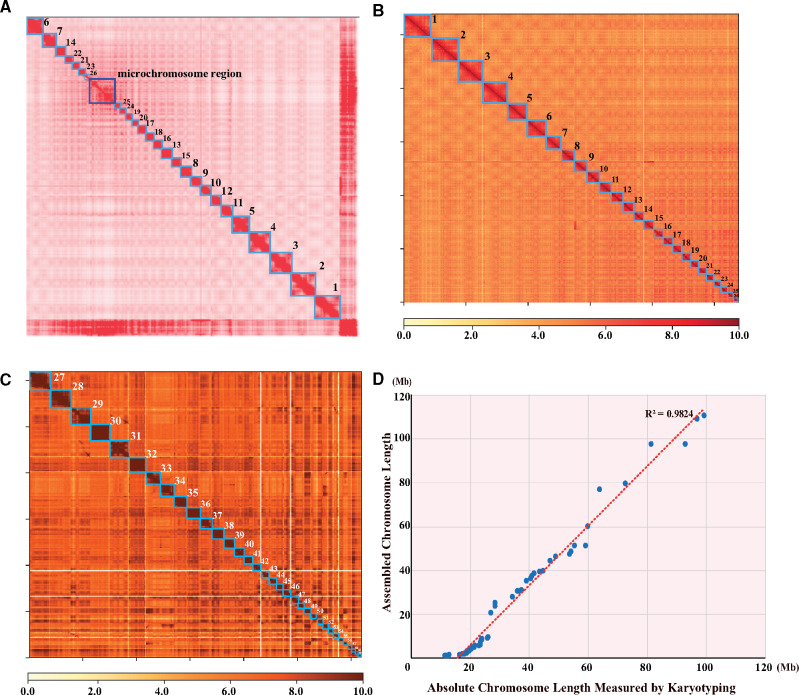
A Hi-C-based chromosome-level genome assembly of the American paddlefish. (*A*) The primary chromosome contact map based on Hi-C data. Each red block in the figure represents clustered chromosome regions with good interactions. Boundaries of the blocks are clear except for the mosaic region. (*B*) The 26 macrochromosomes contact map based on extracted Hi-C data. (*C*) The 34 microchromosomes contact map based on extracted Hi-C data from the mosaic region. (*D*) Strong correlation of chromosome lengths between the Hi-C-based assembly and a previously reported karyotypic analysis (*R*^2^ = 0.9824).

We applied Hi-C technology to construct the chromosomes of the American paddlefish on the basis of the final assembly. A total of 99.3 Gb of raw reads was produced from the BGISEQ500 platform and aligned to the assembled contigs after filtration. The contact count among each contig was calculated and normalized (fig. 1). According to a previous report (Symonová et al. 2017), we set the chromosome number to be 60 pairs (2*n* = 120). Strangely enough, the aligned contigs were anchored into only 26 chromosomes instead, along with a mosaic region on the chromosome contact map (fig. 1*A*). Considering the fact that the American paddlefish genome contains 26 pairs of macrochromosomes, we assumed that these 26 distinguishable clusters with clear boundaries on the contact map (fig. 1*B*) should be macrochromosomes (numbering Chr1 to Chr26), whereas the ambiguous mosaic region (fig. 1*C*) was supposed to contain all microchromosomes, which were too short to be clearly distinguished (fig. 1*A*).

In order to test our hypothesis, we extracted the 26 distinguishable regions in those scaffolds with the clustering, ordering, and orientating information to be reassembled from the previous genome assembly. Interestingly, these putative macrochromosomes (fig. 1*B*) ranged from the smallest Chr26 (20.87 Mb, 1.36% of the genome) to the largest Chr1 (110.67 Mb, 7.18% of the genome). The total length of these macrochromosomes was about 1.34 Gb, occupying 87.05% of the total genome assembly (supplementary table S8, Supplementary Material online). Subsequently, after extraction of these putative macrochromosomes, the remaining sequences of the clustered scaffolds, assumed to be microchromosomes in the mosaic region (fig. 1*A*), were obtained and sorted to construct an additional contact map (fig. 1*C*). As expected, we distinctly identified 34 clusters with clear boundaries representing the 34 short microchromosomes (numbering Chr27 to Chr60) with lengths ranging from 1.18 Mb (Chr60, 0.076% of the genome) to 9.65 Mb (Chr27, 0.631% of the genome). The 34 microchromosomes accounted for only 9.48% (145 Mb in total) of the assembled genome (supplementary table S8, Supplementary Material online).

Evidently, we successfully assembled all the 60 chromosomes of the American paddlefish haploid genome for the first time. The contig and scaffold N50 values of the final chromosome-level genome assembly reached 3.4 and 48.9 Mb, respectively (supplementary table S2, Supplementary Material online). Interestingly, the macrochromosomes had a lower gene density than the microchromosomes (supplementary fig. S3*A* and *B*, Supplementary Material online) due to more exons in each gene and larger intron sizes (supplementary fig. S3*C*, Supplementary Material online). The sequence lengths of our assembled 60 chromosomes and the physical chromosomal size measured by karyotype (Symonová et al. 2017) were highly correlated (*R*^2^ = 0.98; fig. 1*D*).

### Genome Evolution

**Fig. 2.**
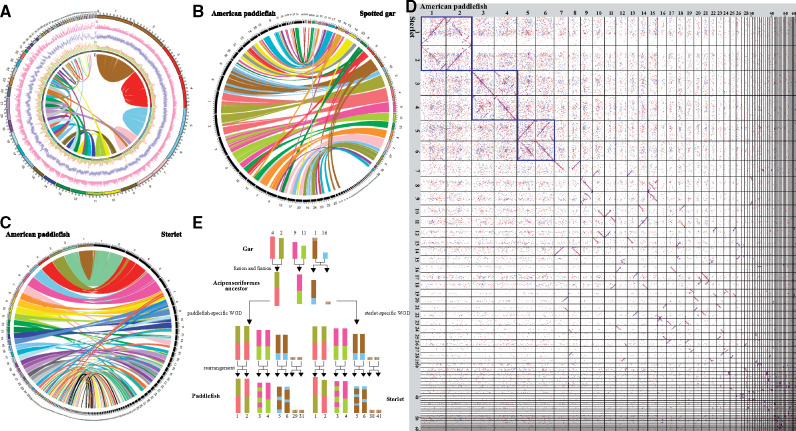
Chromosomal evolution of American paddlefish. (*A*) Intraspecific chromosome comparison within American paddlefish. From outside to inside: (a) chromosome number, (b) gene distribution, (c) Repeat distribution, (d) GC content distribution, and (e) Synteny links. (*B*) Interspecific chromosome comparison between American paddlefish and spotted gar. The left black columns represent the 60 chromosomes of the American paddlefish, and the right colored columns represent the 29 chromosomes of the spotted gar. (*C*) Interspecific chromosome comparison between American paddlefish and sterlet. The left colored columns represent the 60 chromosomes of the American paddlefish, and the right black columns represent the 60 chromosomes of the sterlet. (*D*) Dotplots for sequence alignments between the chromosomes of American paddlefish and the corresponding chromosomes of sterlet (sorting from the longest Chr1 to the shortest Chr60). (*E*) Deduced ancestral chromosomes of the Acipenseriformes.

To study the potential evolutionary pattern of American paddlefish chromosomes, we primarily performed intraspecific chromosomal comparison. We observed that the majority of the chromosomes had synteny blocks (≥2 kb) with the other chromosomes, except for several microchromosomes (fig. 2*A* and supplementary fig. S6, Supplementary Material online), similar to the previously reported sterlet genome (Du et al. 2020). It is noteworthy that the three longest pairs of macrochromosomes (Chr1 and Chr2, Chr3 and Chr4, Chr5 and Chr6) had nearly full and exclusive coverage with each other, whether in the same or the opposite order (supplementary fig. S4*A*–*C*, Supplementary Material online), suggesting that each pair of the macrochromosomes were homologous, possibly caused by a lineage-specific WGD event (Crow et al. 2012). Chr7 and Chr8 showed homology over a majority of their length, although not the full lengths (supplementary fig. S4*D*, Supplementary Material online). Furthermore, each of the other macrochromosomes (Chr7 to Chr26) shared duplicated regions with two or more chromosomes (fig. 2*A* and supplementary fig. S5*A*, Supplementary Material online); this phenomenon occurred in most macrochromosomes and several microchromosomes (such as Chr27, Chr28, Chr39, and Chr43; supplementary fig. S5*B*, Supplementary Material online) as well. Half of the microchromosomes (Chr30, Chr32, Chr36, Chr38, Chr41, Chr45, Chr46, Chr47, Chr49, Chr51, Chr53, and Chr55–Chr60) had completely lost their homologous counterparts (supplementary fig. S6, Supplementary Material online). However, some of the sequences could be aligned to the genome of the spotted gar and/or the sterlet (fig. 2*B*–*D*), suggesting that genes located on these microchromosomes are shared among the fishes and are possibly functional.

Previous studies verified that the spotted gar owned very conserved chromosomes in comparison to other model vertebrates (Braasch et al. 2016); we thus aligned our assembled American paddlefish genome against the chromosomes of the spotted gar to explore potential chromosomal rearrangements. Based on our interspecific comparisons, we observed that most regions in the macrochromosomes and some of the microchromosomes of the American paddlefish could be localized onto those of the spotted gar (fig. 2*B*). Most gar chromosomes have two counterparts in paddlefish, similar to the chromosomal comparison between the gar and sterlet (fig. 2*C*). More specifically, the three longest pairs of macrochromosomes of the American paddlefish could be aligned to the three corresponding pairs of gar chromosomes (LG2 and LG4, LG9 and LG11, LG1 and LG16). For example, gar LG2 and LG4 fused head-to-head to form paddlefish Chr1, and also to form the duplicated Chr2 generated from WGD. Similarly, Chr3/Chr4 was a fusion of gar LG9 and LG11, followed by intrachromosomal rearrangements. Interestingly, gar LG1 and LG16 fused to paddlefish Chr5/Chr6, followed by gar LG1 undergoing fission to form the microchromosome Chr29/Chr31 (fig. 2*E* and supplementary fig. S9, Supplementary Material online). Depending on the conserved status of the spotted gar, we speculate that the American paddlefish may have experienced extensive chromosomal rearrangements during its evolution.

Since both the American paddlefish and the sterlet have homologous chromosomes within each of their own genomes, we aligned both genomes again to verify synteny sequences in each pair of chromosomes between the two species. Dotplots showed that the two genomes were homologous to some extent, both along the macrochromosomes (supplementary fig. S7, Supplementary Material online) and microchromosomes (supplementary fig. S8, Supplementary Material online), especially for the six largest pairs of macrochromosomes (the left top squares in fig. 2*D*). Combined with above intraspecific findings, it seems that although independent lineage-specific WGD events happened after their divergence, the American paddlefish and the sterlet still shared certain common evolutionary patterns in their chromosomes and genome sequences.

### Phylogeny and Divergence Time of Species and Chromosomes

**Fig. 3.**
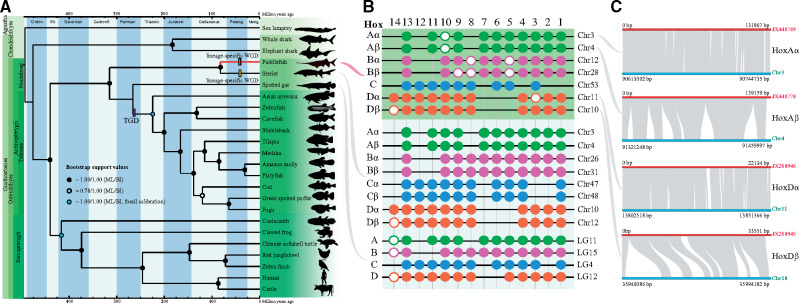
Phylogeny of the American paddlefish and identification of the complete *Hox* clusters. (*A*) The fossil-calibrated phylogenetic tree of 24 examined vertebrates. The ML (maximum likelihood) and BI (Bayesian inference) node supports are presented as filled circles (equal to 1) or hollow circles (less than 1). (*B*) Complete *Hox* clusters in American paddlefish, sterlet, and spotted gar. (*C*) Synteny regions between the assembled chromosomes and previously reported BAC clones of *Hox* clusters.

To estimate the phylogenetic relationship of the paddlefish and sterlet in relation to other vertebrates, we selected 702 single copy orthologous genes in 24 species, totaling 1,475,187 aligned sites (supplementary fig. S10 and table S9, Supplementary Material online). The deduced phylogenetic topology (fig. 3*A*) for each first site codon was solid, as evidenced by the high branch supports (fig. 3*A* and supplementary figs. S11 and S12, Supplementary Material online). The complete coincidence of phylogenetic topologies between the Bayesian inference (BI) and the maximum likelihood (ML) methods suggested high confidence in our reconstructed evolutionary tree. The phylogenetic tree sheds light on the evolutionary status of the American paddlefish, which was placed in the most basal position in the ray-finned fishes. Thus, our phylogenetic analysis from the genome level supported the primitive position of paddlefishes, as in previous researches (Crow et al. 2012; Hughes et al. 2018).

Based on the calibrated nodes in the phylogenetic tree (fig. 3*A* and supplementary fig. S13, Supplementary Material online), we estimated that the lineage of Acipenseriformes represented by the American paddlefish and the sterlet occurred during the Carboniferous at about 314.9 Ma, with a 95% confidence interval ranging from 245.3 to 376.4 Ma (supplementary fig. S13, Supplementary Material online). The two families in Acipenseriformes diverged around 81.5 Ma.

We also performed similar time-calibrated phylogenetic studies to estimate the divergence time of each pair of the identified homologous macrochromosomes of the American paddlefish. Our results showed that Chr1 and Chr2 diverged about 45.6 Ma (supplementary fig. S14, Supplementary Material online), whereas Chr3 and Chr4 (supplementary fig. S15, Supplementary Material online) and Chr5 and Chr6 (supplementary fig. S16, Supplementary Material online) split around 46.6 and 54.1 Ma, respectively. It seems that the sturgeon-specific WGD event happened more recently than the TGD, although a consensus of the exact time has not been reached yet (Crow et al. 2012; Cheng et al. 2019; Du et al. 2020). Our findings from the present study provide additional evidence for such a recent event.

### Prediction of Complete *Hox* Clusters

A total of 75 *Hox* genes distributed in seven clusters were identified from the American paddlefish genome. The two complete *HoxA* clusters were mapped onto Chr3 and Chr4, whereas the two *HoxD* clusters were localized onto Chr10 and Chr11 (fig. 3*B* and *C*). We also identified two *HoxB* clusters and one *HoxC* cluster on Chr12, Chr28, and Chr53 (fig. 3*B*).

To further evaluate the accuracy of our assembly, we determined that the previously published four BAC clones of *Hox* clusters (Crow et al. 2012) displayed a high degree of coverage with our present chromosome-level assembly (fig. 3*C*). In detail, 100%, 98.7%, 89.1%, and 100% of the sequences from BAC352P4 (*HoxAα*), BAC370N10 (*HoxAβ*), BAC231C24 (*HoxDα*), and BAC249G23 (*HoxDβ*) were covered, respectively. The high coverage between our data and these previously reported clones supports the high reliability of our chromosome-level assembly for the American paddlefish.

### *SCPP* Genes Uncovered in the Early Vertebrates

**Fig. 4.**
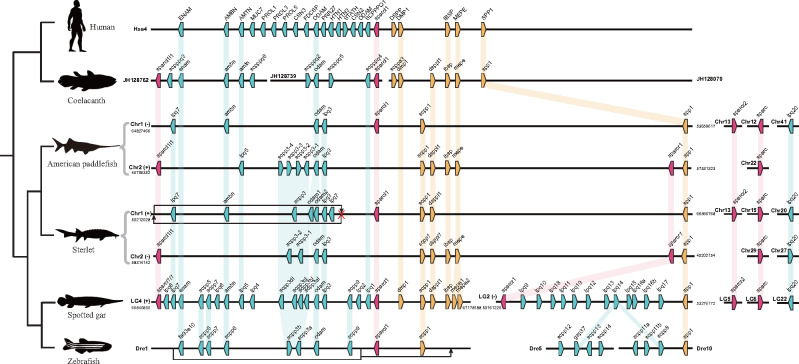
*SCPP* gene arrangement in paddlefish (this study), sterlet, and other vertebrates. P/Q-rich *SCPP* genes and acidic *SCPP* genes are marked by blue and yellow pentagons, respectively; *sparcl1*, *sparcl1l1*, and *sparcr1* are marked by red pentagons. Orthologs are linked with shadows. Note that there is a reverse of P/Q-rich genes on Chr1 in the sterlet genome, and this region is located downstream of *scpp1* on Dre1 of the zebrafish.

**Fig. 5.**
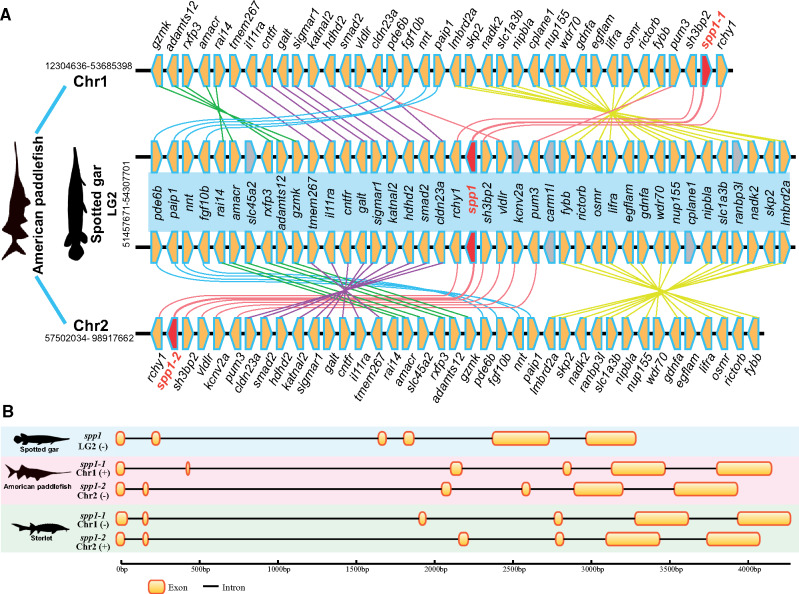
Identification of two *spp1* (*spp1-1* and *spp1-2*) genes and genomic comparison of neighboring genes in American paddlefish and spotted gar. Each polygon marks to a gene (*A*). The yellow polygons represent hits between the spotted gar and American paddlefish, whereas the gray polygons indicate no hits. The red polygons are *spp1* genes. Gene structures of the identified *spp1* genes in American paddlefish and sterlet are provided (*B*). Each yellow box represents an exon.

Paddlefishes and sturgeons are good models for studying bone mineralization, since they retain a relatively primitive phenotype but have derived cartilaginous skeletons (as in sharks) despite their ancestors having bony skeletons (Helfman et al. 2009).Spotted gar seems to have the largest number of bone mineralization-related *SCPP* genes (38 in total) identified to date (Braasch et al. 2016; Kawasaki et al. 2017), which is reasonable since it has ganoid scales, heavily ossified bones, and a full set of teeth. In the present study, we identified 25 and 27 *SCPP* genes (including ancient *SPARC* genes) in the American paddlefish and the sterlet, respectively (fig. 4). In further BLAST searching of 40 nearby genes of *spp1* with a genomic spanning of about 3 Mb in the spotted gar genome (supplementary table S10, Supplementary Material online) against the assembled chromosomes of the American paddlefish, we identified 36 and 38 genes neighboring *spp1-1* and *spp1-2* genes with high correlations (fig. 5), strongly indicating the existence of two putative *spp1* genes in the American paddlefish genome. Two *spp1* sequences with conserved RGD motif (an integrin-binding Arg–Gly–Asp motif) were also successfully cloned from the paddlefish genomic DNA (fig. 5 and supplementary fig. S17 and table S11, Supplementary Material online). Our results indicated that, unlike the role *spp1* plays in shark and zebrafish (Venkatesh et al. 2014), other members in *SCPP* family or even other gene families might be involved in the reversion from a bony to cartilaginous feature of the paddlefishes and sturgeons.

## Discussion

### Resolution of a Complex Chromosome-Level Genome Assembly Using Hi-C Data

In this study, we have provided a model and an example of using Hi-C data to assemble a complex fish genome with a large number of variable chromosomes. The American paddlefish genome contains 120 chromosomes (Symonová et al. 2017), and thus it was a formidable challenge to perform a cytogenetic analysis. A karyotypic test estimated that the genome consists of 48 macrochromosomes and 72 microchromosomes (Dingerkus and Howell 1976). Another more recent study with cytogenetic markers suggested that there were 54 macrochromosomes and 66 microchromosomes in the American paddlefish (Symonová et al. 2017). In these studies, however, the boundary between macrochromosomes and microchromosomes seems to be unclear.

Our present chromosome-level assembly based on additional Hi-C data showed that the haploid paddlefish genome comprised 26 identifiable macrochromosomes and 34 microchromosomes (fig. 1), which is very close to the estimated 54 + 66 (2*n*) chromosomes from the previous karyotypic analysis, and the lengths of the assembled chromosomes were highly correlated with the measured physical sizes (Symonová et al. 2017). The overall similarity in both size and number between the Hi-C assembled and physically tested genomes confirmed the existence of both macro- and microchromosomes in the American paddlefish, which is also a shared feature in the genomes of sturgeons (Du et al. 2020).

The present study provides a practical solution for any chromosome-level assembly of a complex fish genome. Our results illustrate the possibility of reconstructing the ancestral Acipenseriformes chromosomes for further understanding the origin of paddlefishes and sturgeons.

### An Interesting Chromosomal Evolution Pattern of the American Paddlefish

In Acipenseriformes, the most distinctive characteristics of karyotypes are the high chromosome numbers (100–360) and the presence of microchromosomes (Symonová et al. 2017). However, the reasons for such a high number are as yet unknown. Recently, the genome assembly of sterlet has shed some light on the mechanisms of segmental rediploidization and chromosomal loss and rearrangement (Du et al. 2020). In the current study, with the intraspecific and interspecific comparisons between the American paddlefish, sterlet, and spotted gar, we delineated possible evolutionary processes of the American paddlefish chromosomes based on the whole-genome comparisons.

In the intraspecific comparisons, many duplicated regions were identified between the chromosomes. However, unlike the obvious one-to-one syntenic relationship of all paired chromosomes in the common carp (Xu et al. 2014), the presence of one-to-one synteny conservation was only observed between the three largest pairs of macrochromosomes (fig. 2*A* and supplementary fig. S4, Supplementary Material online), validating the lineage-specific WGD event in the American paddlefish (Symonová et al. 2017). In addition, each pair of these paralogous chromosomes has similar repeat content, showing no evidence for allopolyploidy (supplementary fig. S18, Supplementary Material online). Extensive interchromosomal changes happened thereafter, but rearrangements mainly occurred on smaller macrochromosomes (Chr7–Chr26).

In the interspecific comparison, American paddlefish displayed an intricate relationship with spotted gar, whose genome has conserved in content and size many entire chromosomes (*n* = 29) from bony vertebrate ancestors (Braasch et al. 2016). Interestingly, the alignment did not clearly reveal an expected one-to-two relationship between the spotted gar and the paddlefish chromosomes, whereas a two-to-two pattern was identified between the two largest pairs of the paddlefish macrochromosomes and the corresponding linkage groups of the spotted gar, possibly due to the fusion of two ancestral chromosomes (fig. 2*E*). Gar LG1 and LG16 can map to paddlefish Chr5 and Chr6, Chr29 and Chr31, showing a two-to-four pattern, which is a consequence of the fusions as mentioned above, followed by a fission of ancestral chromosome related to gar LG1, leading to the formation of paired microchromosomes in the American paddlefish. Furthermore, this chromosomal evolution pattern was also found in the sterlet, and helped us to deduce the Acipenseriformes ancestral chromosomes, which include large macrochromosomes fused from two ancient chromosomes and microchromosomes that had been fissioned from a single chromosome (fig. 2*E*).

Interspecies chromosomal comparison between American paddlefish and sterlet shows homology between the two fish species (fig. 2*C* and *D*). Not only macrochromosomes (supplementary fig. S7, Supplementary Material online) but also microchromosomes (supplementary fig. S8, Supplementary Material online) were highly conserved in some regions along the chromosome, confirming the low evolutionary rate of Acipenseriformes species (Du et al. 2020). Similar to the sterlet, the American paddlefish also had chromosome losses and rearrangements (fig. 2*A* and supplementary figs. S4–S6, Supplementary Material online) that may provide a reasonable explanation for the same mechanisms of segmental rediploidization and the evolving of microchromosomes among various species in Acipenseriformes (Symonová et al. 2017; Du et al. 2020).

Therefore, taking these genomic comparisons into consideration, we hypothesize that there were extensive chromosomal rearrangements in the American paddlefish both before and after the WGD event.

### Phylogeny and Divergence Time of the American Paddlefish and Chromosomes

Paddlefishes have retained some primitive characteristics, including the skeleton, heterocercal fins, and body shape. Previous molecular studies based on single or multiple mitochondrial or nuclear gene(s) supported a basal phylogenetic position of Actinopterygii (Hughes et al. 2018). Our present data based on orthologs from whole genomes further validated this basal status in Actinopterygii. Meanwhile, the phylogenetic branch of the American paddlefish presented a similar length to that of the sterlet, suggesting a similar slow evolutionary rate as previously estimated in the sterlet (Du et al. 2020) that are comparable to the spotted gar, which was considered as the most slowly evolved fish except for the coelacanth (Braasch et al. 2016). It seems that the slow evolutionary rate is consistent with the morphological conservation in the American paddlefish. With fossil-calibrated dating of the whole-genome orthologs-based phylogeny, we estimated that the ancestor of paddlefishes and sturgeons originated about 314.9 Ma, and this is consistent with previous molecular studies (Hughes et al. 2018).

Time-calibrated phylogenies of each pair of the identified homologous macrochromosomes revealed a relatively recent WGD event in the American paddlefish about 46.6–54.1 Ma, consistent with the previous estimate of about 42.7 Ma based on the *HoxA* gene cluster (Crow et al. 2012). However, this estimate might be quite far off the time when the event actually happened due to delayed rediploidization (Robertson et al. 2017). Nonetheless, it is earlier or much later than the reported 21.3 Ma (Cheng et al. 2019) or 180 Ma (Du et al. 2020) of the sterlet WGD. Thus, it is necessary to carry out more analyses to confirm the exact date of the independent WGD events in the two families within the Acipenseriformes.

In addition, all three topologies support the divergence of species before the divergence of each pair of the identified homologous chromosomes, suggesting that the WGDs of the paddlefish and sterlet were two independent events. Additional 4dTv analysis also shows two different peaks for the two species, indicating different occurrence times of the two WGDs (supplementary fig. S19, Supplementary Material online). However, due to the limitations of both phylogenetic and 4dTv analyses, the current results cannot rule out a shared WGD.

### *SCPP* Genes in the American Paddlefish

The discovery of *SCPP* genes in paddlefish and sterlet uncovers the earliest flourishing of this family occurred at least before the split between Acipenseriformes and teleost. *SCPP* genes can be classified into two groups. The acid genes are involved in formation of bone and/or dentin, and the Pro/Gln (P/Q)-rich genes are related to formation of enamel or enameloid matrix, mostly expressed in skin and scales (Kawasaki et al. 2017). Paddlefish and sterlet retain most of the acid *SCPP*s except for *dmp1*, a gene that functions in the mineralization of bone and dentin (Ling et al. 2005). This might be one cause for the special cartilaginous phenotype of Acipenseriformes fishes. However, these fishes had fewer P/Q-rich *SCPP*s compared with spotted gar (fig. 4). It seems that they lost the whole cluster of P/Q-rich genes (mainly expressed in skin and scales, but not in teeth or bone) between *sparcr1* and *spp1* as in tetraploids, suggesting that the cluster may have been first derived in the spotted gar. In the other cluster adjacent to *sparcl1*, some genes were lost but some were retained. For example, the gene *enam*, crucial for formation of the enamel matrix of teeth (Deméré et al. 2008), has been lost in the toothless paddlefishes and sturgeons but exists in vertebrates with teeth (such as human, coelacanth, spotted gar, and zebrafish; fig. 4). In addition, both American paddlefish and sterlet apparently retained only one copy of the ancient *sparc* genes (*sparcl1l1*, *sparcl1*, and *sparcr1*) after the genome duplication, although one or more were lost in tetrapods and teleosts (fig. 4). Therefore, it is possible that nonteleost ray-finned fishes may retain the largest number of ancient *sparc* genes.

As an acidic member of the *SCPP* family, *spp1* is mainly related to tissue mineralization such as during tooth formation, bone formation, and potential scale formation (Kawasaki et al. 2017). Many reports have shown that *spp1* may play an essential role in bone formation in zebrafish, leading to the hypothesis that absence of *spp1* could be accountable for the cartilaginous skeleton in Chondrichthyes (Venkatesh et al. 2014; Kawasaki et al. 2017). Our data strongly suggest the existence of two *spp1* copies in the American paddlefish (and the sterlet), indicating that the hypothesis of *spp1*’s responsibility for cartilaginous features may be incompatible with the American paddlefish.

## Conclusions

Research on sturgeons and paddlefishes has long been a hot topic due to the special evolution, economic importance, and endangered status of these fishes. However, genomic studies have been greatly hampered by the extreme complexity of these genomes with high chromosome numbers and various macro-/microchromosomes. Here, we provided the first chromosome-level genome assembly of the American paddlefish in the Acipenseriformes. The success of assembling 26 macrochromosomes and 34 microchromosomes in the haploid genome indicates that extensive chromosomal rearrangements, including fusions to form the macrochromosomes and fissions to form the microchromosomes, have occurred in this ancient fish. Most acid *SCPP* genes were retained but some P/Q-rich genes were lost in the American paddlefish, providing new insights into the mineralization of bones, teeth, and scales of the early vertebrates.

## Materials and Methods

### Fish Collection and Species Identification

An artificially cultivated American paddlefish (about 5 years old, 1 m in snout-tail length, 3.5 kg in body weight) was sampled from a local hatchery in Taihu Station, Yangtze River Fisheries Research Institute (YFI), Chinese Academy of Fisheries Sciences (CAFS), Wuhan City, Hubei Province, China. The fish was identified on the basis of both DNA barcoding (*COI* gene sequence) and morphological observation. All the fish handling and experimental procedures used in this study were approved by the Animal Care and Use Committee of the YFI of CAFS, China (Animal Welfare Assurance No. YF001).

### DNA/RNA Extraction and Sequencing

Genomic DNA samples from either blood or muscle were collected from the same fish for whole-genome sequencing with standard protocols. We employed the routine whole-genome shotgun-sequencing strategy (Venter et al. 2001) to construct three short-insert (270, 500, and 800 bp) and four long-insert (2, 5, 10, and 20 kb) libraries, according to standard protocols from Illumina (San Diego, CA). Paired-end (PE) sequencing was carried out on an Illumina HiSeq 2500 platform (blood sample; PE125 for 270-, 500-, and 800-bp libraries) and HiSeq X Ten platform (muscle sample; PE150 for the remaining DNA libraries). Low-quality raw reads (more than 10 Ns, or rich in low-quality bases) were removed by SOAPfilter version 2.2 with optimized parameters (-y -p -g 1 -o clean -M 2 -f 0).

Additional blood samples were collected for genomic DNA extraction using the traditional phenol/chloroform extraction method to perform PacBio long-read sequencing as reported in a previous study (Jiang et al. 2019). High-quality DNA was used to construct a SMRATbell library with an insert size of 30 kb and sequenced on a PacBio Sequel platform (Pacific Biosciences, Menlo Park, CA).

To achieve an updated chromosome-level assembly, we applied the Hi-C method (Burton et al. 2013) to detect chromatin interactions in the American paddlefish nucleus. First, we utilized the restriction enzyme *Mbo*I to digest genomic DNAs from blood tissue after conformation fixing by formaldehyde and repaired 5′ overhang using biotinylated residue. After ligation of blunt-end fragments in situ, the isolated DNAs were reverse-cross-linked, purified, and filtered for biotin-containing fragments. Subsequently, DNA fragment end repair, adaptor ligation, and PCR were performed, and a 400-bp insert library was constructed for sequencing on a BGISEQ-500 platform (BGI, Shenzhen, China) to generate short paired-end reads with a length of 100 bp (Huang et al. 2017).

For gene annotation of the assembled genome, transcriptome sequencing was performed with blood tissue from the same American paddlefish. Total RNA was extracted with TRIzol Reagent (Invitrogen, Carlsbad CA). A Nanodrop ND‐1000 spectrophotometer (LabTech Int, East Sussex, UK) and a 2100 Bioanalyzer (Agilent Technologies, Palo Alto, CA) were used to check RNA quality, and two micrograms of verified RNAs were used for library construction and transcriptome sequencing on an Illumina HiSeq 4000 platform.

### Genome Size Estimation and De Novo Genome Assembly

Genome size of the American paddlefish was estimated based on the routine 17-mer depth frequency distribution analysis (Liu et al. 2013) using the short reads from the above-mentioned 500- and 800-bp Illumina libraries.

Subsequently, a de novo genome assembly was generated using both the Illumina short reads and PacBio long reads. First, the Illumina short-insert (270, 500, and 800 bp) sequencing data were assembled into contigs with optimized parameters (-k 29 -d 0.3 -t 16 -m 300) by Platanus version 1.2.4 (Kajitani et al. 2014). The initial contigs were aligned against the PacBio long reads by DBG2OLC (Ye et al. 2016) to obtain consensus sequences that were further polished by Pilon version 1.22 (Walker et al. 2014). Next, PacBio reads were used to construct the primary scaffolds by SSPACE-LongRead (Boetzer and Pirovano 2014) based on the polished contig assembly. Illumina long-insert (2, 5, 10, and 20 kb) sequencing data were then used to connect the obtained scaffolds by SSPACE_Standard version 3.0 (Boetzer et al. 2011). Gaps within these scaffolds were eventually filled by GapCloser version 1.12 and GapFiller version 1.10 (Nadalin et al. 2012), and the obtained scaffolds were polished by Pilon (Walker et al. 2014) again to generate the final genome assembly of the American paddlefish. Completeness of the draft genome assembly was evaluated using BUSCO version 3.0.2 (Simão et al. 2015) with default parameters (-m genome -l actinopterygii_odb9 -c 8 -f -e 0.01).

### Construction of a Chromosome-Level Genome Assembly Using the Hi-C Technology

Hi-C raw data were first mapped to our genome assembly of the American paddlefish to remove nonmapped, duplicated, and invalid reads, with the remaining valid pairs of reads accepted by HiCPro version 2.2 (Servant et al. 2015) for further analysis.

A chromosome contact matrix was constructed using interaction frequencies, which were calculated from the number of the Hi-C paired-end reads mapped to the generated scaffolds. All interactions were clustered from the chromosome contact matrix. An original chromosome contact map displaying sequence clustering was generated and an “AGP” (A Golden Path) file with both the position and direction of all clustered sequences was created by Juicer version 1.5 (Durand et al. 2016). In this step, we temporarily assigned the chromosome number as 60 pairs (2*n* = 120) based on previous studies (Symonová et al. 2017).

According to the chromosome contact map, we identified the boundaries of each clustering block and manually checked the validity in the “AGP” file. Sequences representing the 26 distinguishable districts on the original map were retrieved from the file to create a contact map for all macrochromosomes. The rest of the sequences, forming a mosaic region on the original map, were applied to construct another contact map for all microchromosomes. In total, 60 pairs of chromosomes of the American paddlefish were fully recovered.

In order to evaluate the accuracy and reliability of our genome assembly, we checked the relationship between the assembled size and physical size (measured by karyotyping; Symonová et al. 2017) of each chromosome. Chromosomes were sorted by length from the shortest to the longest, and a correlation map was created to show their consistency. We also applied previously published short assemblies (Crow et al. 2012) of two *HoxA* clusters (BAC352P4: GenBank accession number JX448769.1, and BAC370N10: number JX448770.1) and two *HoxD* clusters (BAC249G23: number JX280945.1, and BAC231C24: number JX280946.1) from the American paddlefish to examine the coverage of our upgraded assembly; the analysis was implemented in Lastz version 1.02 (Harris 2007) with optimized parameters of “*T* = 2 *C* = 2 *H* = 2,000 *Y* = 3,400 *L* = 6,000 *K* = 2,200.”

### Repeat Element Annotation

De novo repeat libraries were initially constructed by RepeatModeller version 1.05 (Maziade et al. 1996) and LTR_FINDER.x86_64 version 1.0.6 (Xu et al. 2007) with default parameters. Subsequently, the draft genome assembly was aligned to RepBase version 21.01 (Jurka et al. 2005), and the de novo repeat libraries were used to identify known and novel transposable elements by RepeatMasker version 4.06 (Graovac and Chen 2009). Meanwhile, tandem repeated sequences were annotated by Tandem Repeat Finder version 4.07 (Benson 1999) with optimized parameters as follows: “Match = 2, Mismatch = 7, Delta = 7, PM = 80, PI = 10, Minscore = 50, and MaxPeriod = 2,000.” Finally, transposable element relevant proteins in our genome assembly were predicted by RepeatProteinMask (Graovac and Chen 2009).

### Gene Prediction and Functional Annotation

Three standard strategies, that is, homology, de novo, and transcriptome-based annotations, were combined to predict a total gene set for the American paddlefish genome.

For the homology annotation, we aligned protein sequences from published genomes (downloaded from NCBI Genome database) of ten representative vertebrates, including elephant shark (*Callorhinchus milii*), zebrafish (*Danio rerio*), medaka (*Oryzias latipes*), fugu (*Takifugu rubripes*), green spotted puffer (*Tetraodon nigroviridis*), pike (*Esox lucius*), stickleback (*Gasterosteus aculeatus*), cod (*Gadus morhua*), sea lamprey (*Petromyzon marinus*), and spotted gar (*Lepisosteus oculatus*), against the genome assembly of the American paddlefish to predict homologous genes. These genes were searched by BLAST (version 2.2.6; mode: TBlastN, Altschul et al. 1990) with an e-value of 10^−5^. The data from BLAST searching were further processed via Sorting Out Local Alignment (Yu et al. 2006) to obtain the best fit of each alignment. Subsequently, gene structures were predicted by GeneWise version 2.2.0 (Birney et al. 2004) from these best hits. Those low-quality predictions (predicted genes with less than 150 bp for the entire length) were removed.

For the de novo annotation, the assembled scaffolds were masked based on the above-mentioned repeat annotation. We applied AUGUSTUS version 2.5 (Stanke et al. 2006) and GENSCAN version 1.0 (Burge and Karlin 1997) for the de novo prediction of repeat-masked genome sequences. Low-quality predictions were also discarded using the same screening threshold as for the homology annotation.

For the transcriptome-based annotation, the blood transcriptome data were mapped onto the assembled scaffolds to identify splice junctions by TopHat version 2.1.1 (Trapnell et al. 2009). These mapped transcriptome reads were then assembled by Cufflinks version 2.2.1 (Trapnell et al. 2010) to assist gene annotation.

Finally, all the above-mentioned gene sets were merged together to yield a comprehensive and nonredundant gene set by utilizing GLEAN (Elsik et al. 2007). To understand the potential functions of the final gene set, we chose four public databases (including Pfam, PRINTS, ProDom, and SMART) to realize functional annotation.

### Chromosomal Intraspecific and Interspecific Comparisons

To understand the evolved chromosomal patterns in the American paddlefish, we performed both intraspecific and interspecific comparisons.

For the intraspecific comparison, we extracted each chromosome from the American paddlefish as the query, and other chromosomes were set as targets for examination. Thus, the pairs of the intraspecific data set were constructed, and each of these pairs was aligned separately. All alignments were realized by Lastz (Harris 2007) with the same parameters “*T* = 2 *C* = 2 *H* = 2,000 *Y* = 3,400 *L* = 6,000 *K* = 2,200,” and those regions over 2,000 bp were regarded as reliable for each alignment. Simultaneously, we applied all-to-all BLAST (BlastP mode) analysis to identify the syntenic regions between each batch of chromosomes, and those blocks with at least 15 genes were selected as reliable alignments.

For the interspecific comparisons, we compared the chromosome-level assembly of the American paddlefish with those of the spotted gar (Braasch et al. 2016) and the sterlet (Du et al. 2020) using the above-mentioned Lastz method (Harris 2007) with the same parameters. To verify the chromosomal evolution pattern, we aligned homologous chromosome pairs within the paddlefish or between the paddlefish and the sterlet using the LAST package (Kielbasa et al. 2011). Dotplots were generated using filtered alignments with an error probability >1e-8.

### Fossil-Calibrated Phylogenetic Analysis

Whole-genome encoding sequences from 24 vertebrate species were selected for phylogenetic analysis. The jawless vertebrate sea lamprey was employed as the outgroup, and the American paddlefish and 22 other species were used as in-group species. These 22 vertebrates included the eight species used for gene prediction (elephant shark, zebrafish, cod, stickleback, spotted gar, medaka, fugu, green spotted puffer) and 14 other vertebrates, including sterlet, whale shark (*Rhincodon typus*), Asian arowana (*Scleropages formosus*), Mexican tetra (*Astyanax mexicanus*), tilapia (*Oreochromis niloticus*), Amazon molly (*Poecilia formosa*), platyfish (*Xiphophorus maculatus*), coelacanth (*Latimeria chalumnae*), clawed frog (*Xenopus tropicalis*), Chinese softshell turtle (*Pelodiscus sinensis*), zebra finch (*Taeniopygia guttata*), red junglefowl (*Gallus gallus*), cattle (*Bos Taurus*), and human (*Homo sapiens*). We utilized BLAST (mode BlastP) to calculate a super similarity matrix for each paired sequence with an E-value threshold of 1e-5. OrthoMCL (Li et al. 2003) was applied to distinguish gene families based on the super similarity matrix, and a Markov Chain Clustering (MCL) with default parameters was assigned. Once one-to-one orthologs were identified, we extracted them and performed a multiple alignment using MUSCLE version 3.7 (Edgar 2004). Subsequently, the protein alignments were converted to corresponding coding sequences (CDS). The nucleotides of the first position in each codon of all coding sequences were chosen for the constitution of a super-length “fake gene” that was used for a phylogenetic analysis with the ML method. The ML method was implemented in PhyML version 3.0 (Guindon et al. 2010) with a gamma distribution across aligned sites and an HKY85 substitution model. The approximate likelihood ratio test (aLRT) was employed to evaluate the branch supports. To further confirm the deduced topology, we simultaneously performed BI using MrBayes version 3.2.2 (Ronquist et al. 2012) with the HKY85 substitution model. We performed two parallel runs of 200,000 generations and sampling every 200 generations. The initial 25% of all the runs was abandoned for unreliability, whereas the remaining samples were used to establish a maximum clade credibility tree.

After the phylogeny construction, we set two fossil-calibrated nodes in the phylogenetic topology to estimate the date of divergence of the American paddlefish from other vertebrates, which was based on the Bayesian method using MCMCtree in PAML version 4.9e (Yang 2007). Two fossil-calibrated nodes (C1 and C2) were considered as normal distributions and soft constraint bands (allowing a small probability [0.025] of violation). The C1 calibration point was estimated to be the most recent common ancestor (MRCA) of Sarcopterygii based on the fossils from *Latimeria* with a hard-minimum age of 408 Ma and a 95% soft maximum age of 427.9 Ma (Benton et al. 2015). The C2 calibration point was estimated as the MRCA of Teleostei from *Danio* with a hard-minimum age of 151.2 Ma and a 95% soft maximum age of 252.7 Ma (Setiamarga et al. 2008). A total of 100,000 samples were used for the Markov Chain Monte Carlo (MCMC) analysis (Ronquist et al. 2012), and the first 20% of the samples were discarded as a burn-in. An independent rate model (clock = 2) following a lognormal distribution was applied for the MCMC search.

To predict the timing of the WGD event in the American paddlefish, we conducted another batch of fossil-calibrated phylogenetic analyses using the same species and method as mentioned above, where the data were limited to the three longest pairs (Chr1–Chr2, Chr3–Chr4, Chr5–Chr6) of the macrochromosomes in the American paddlefish and the sterlet, along with the whole-genome sequences of the remaining selected species. The divergence times of the chromosomes were estimated by calibrating the tree using the same fossils as mentioned above (Setiamarga et al. 2008; Benton et al. 2015).

### Characterization of *SCPP* Genes and Complete *Hox* Clusters

Elephant shark, whale shark, American paddlefish, and sterlet have a shared cartilaginous and low-mineralized bone feature. Therefore, with protein sequences encoded by 38 *SCPP* mineralization-related genes (seven encode “acidic residue-rich” proteins and 31 encode “Pro/Gln (P/Q) rich” proteins) from spotted gar (Kawasaki et al. 2017) as the queries, we first performed BlastP searches separately against the genomes of the American paddlefish and the sterlet, and then extracted the exon sequences using Exonerate (Slater and Birney 2005). Subsequently, the ancient *sparc* genes (*sparcl1, sparcl1l1*, and *sparcr1* from which *SCPP* genes were derived) were also studied via the same method, using sequences from the spotted gar as references (Kawasaki et al. 2017). One important gene, *spp1*, reported to be missing in sharks (Kawasaki et al. 2017), was cloned experimentally using PCR as an example to verify the results predicted from the assembled genome.

In addition, we searched protein sequences of *spp1* and 40 neighboring genes from the spotted gar to detect the syntenic correlations to American paddlefish. Gene searching was performed by BLAST (BlastP mode, Altschul et al. 1990) with 20 genes in the 5′ adjacent region of *spp1* (including *pade6b*, *paip1*, *nnt*, *fgf10b*, *rail4*, *amacr*, *slc45a2*, *rxfq3*, *adamts12*, *gzmk*, *tmem267*, *il11ra, cntfr*, *galt*, *sigmar1*, *katnal2*, *hdhd2*, *smad2*, *cldn23a*, and *rchy1*) and another 20 genes in the 3′ adjacent region (*sh3bp2*, *vldlr*, *kcnv2a*, *pum3*, carm1l, *fybb*, *rictorb*, *osmr*, *lifra*, *egflam*, *gdnfa*, *wdr70*, *nup155*, *cplane1*, *nipbla*, *slc1a3b*, *ranbp3l*, *nadk2*, *skp2*, and *lmbrd2*) in the spotted gar. According to the differences of bone features between the three fish species along with spotted gar and zebrafish (with highly mineralized bones), we speculate the possible early evolution of *spp1* in an attempt to discern whether the previous hypothesis of *spp1* absence for the cartilaginous feature (Deméré et al. 2008) is suitable for the American paddlefish.

In addition to the two reported complete *HoxA* and two partial *HoxD* clusters (Crow et al. 2012), we attempted to characterize the complete set of *Hox* clusters in the American paddlefish genome. First, we downloaded the complete *Hox* cluster sequences from the spotted gar (Braasch et al. 2016) and the sterlet (Cheng et al. 2019; Du et al. 2020). Then, the obtained protein sequences were BLAST (TBlastN mode) searched against our genome assembly, and the aligned sequences were further verified by Exonerate (Slater and Birney 2005).

## Supplementary Material

Supplementary data are available at *Molecular Biology and Evolution* online.

## Supplementary Material

msaa326_Supplementary_Data
