# Supplementary material for: The American Paddlefish Genome Provides Novel Insights into Chromosomal Evolution and Bone Mineralization in Early Vertebrates
Source: Mol Biol Evol. 2020 Dec 17;38(4):1595–607. doi: 10.1093/molbev/msaa326 (PMC8042750; doi:10.1093/molbev/msaa326)
Supplement: msaa326_Supplementary_Data [file msaa326_supplementary_data.pdf]

Articles: Discoveries

Supplementary Materials for

The American paddlefish genome provides novel insights into chromosomal evolution and bone mineralization in early vertebrates

[1]Peilin Cheng†, [2]Yu Huang†, [2,5]Yunyun Lv†, [1]Hao Du, [2]Zhiqiang Ruan, [1]Chuangju Li, [1]Huan Ye, [1]Hui Zhang, [1]Jinming Wu, [1]Chengyou Wang, [1]Rui Ruan, [2,5]Yanping Li, [2]Chao Bian, [2]Xinxin You, [3]Chengcheng Shi, [3]Kai Han, [2]Junming Xu, [2,4]Qiong Shi*, [1]Qiwei Wei*.

[1]Key Laboratory of Freshwater Biodiversity Conservation, Ministry of Agriculture and Rural Affairs of P. R. China, Yangtze River Fisheries Research Institute, Chinese Academy of Fishery Sciences, Wuhan 430223, China.

[2]Shenzhen Key Lab of Marine Genomics, Guangdong Provincial Key Lab of Molecular Breeding in Marine Economic Animals, BGI Academy of Marine Sciences, BGI Marine, BGI, Shenzhen 518083, China.

[3]BGI-Qingdao, BGI-Shenzhen, Qingdao 266555, China.

[4]Laboratory of Marine Genomics, School of Life Sciences and Oceanography, Shenzhen University, Shenzhen 518060, China.

[5]Key Laboratory of Sichuan Province for Fishes Conservation and Utilization in the Upper Reaches of the Yangtze River, Neijiang Normal University, Neijiang 641000, China.

†These authors contributed equally to this work.

*Correspondence: Email: shiqiong@genomics.cn (Q.S.); weiqw@yfi.ac.cn (Q.W.), Tel.: +86-185 6627 9826 (Q.S.), +86-180 8600 6888 (Q.W.)

This file includes:

Figures S1 to S19

Tables S1 to S11

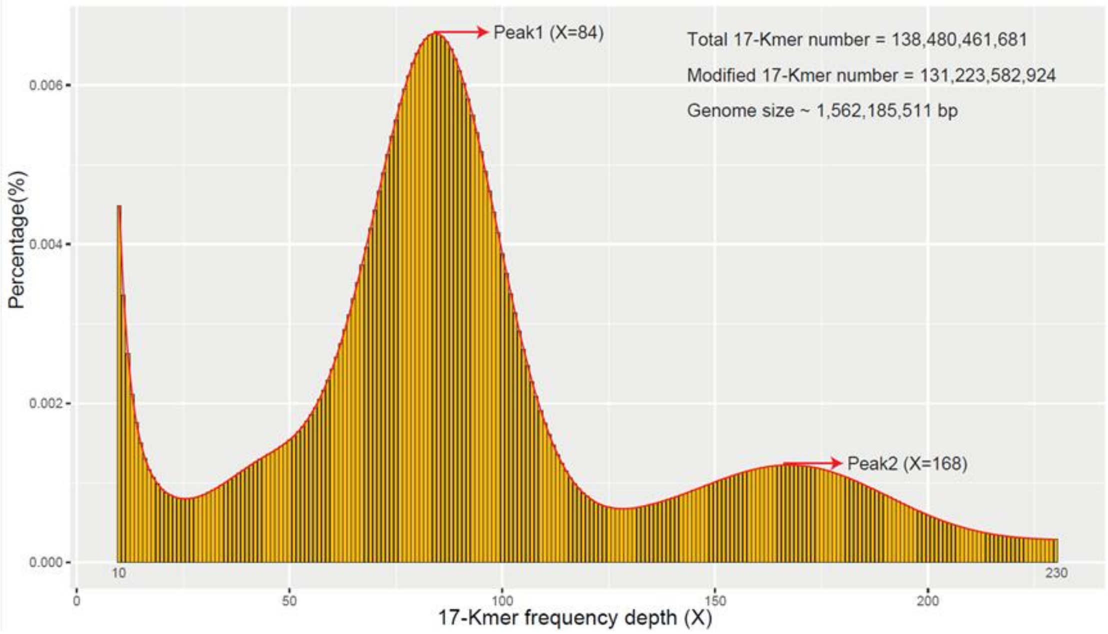

Figure S1. Genome survey of the American paddlefish using a 17-mer analysis. The peaks of homozygous and repeated 17-mers are highlighted. The highest peak (Peak1, X=84) was used to estimate the genome size.

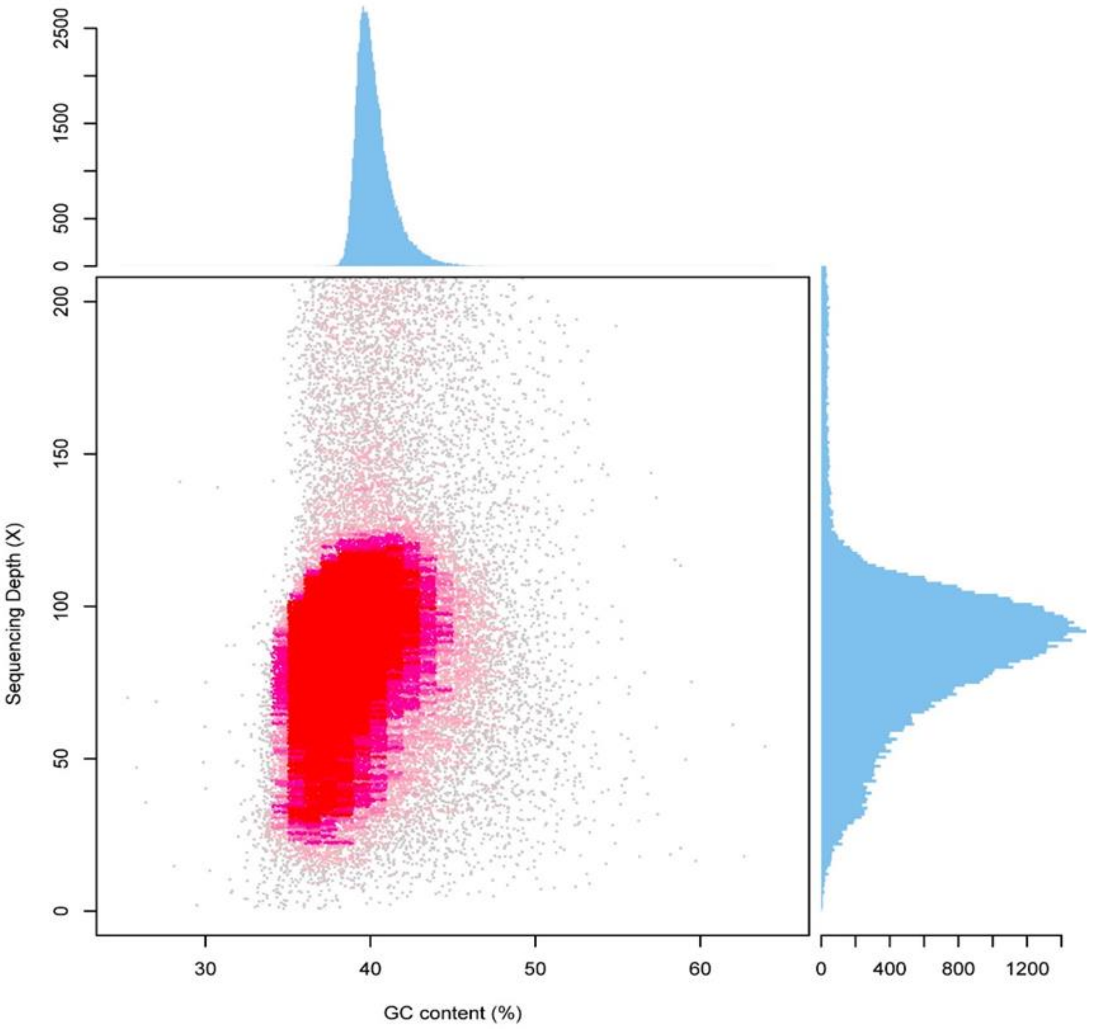

Figure S2. GC content and sequencing depth of the scaffold assembly. In the main plot, the X-axis and Y-axis represent GC content proportion and average sequencing depth, respectively. The right and upper bar charts show the numbers of hits at the given sequencing depth or GC content.

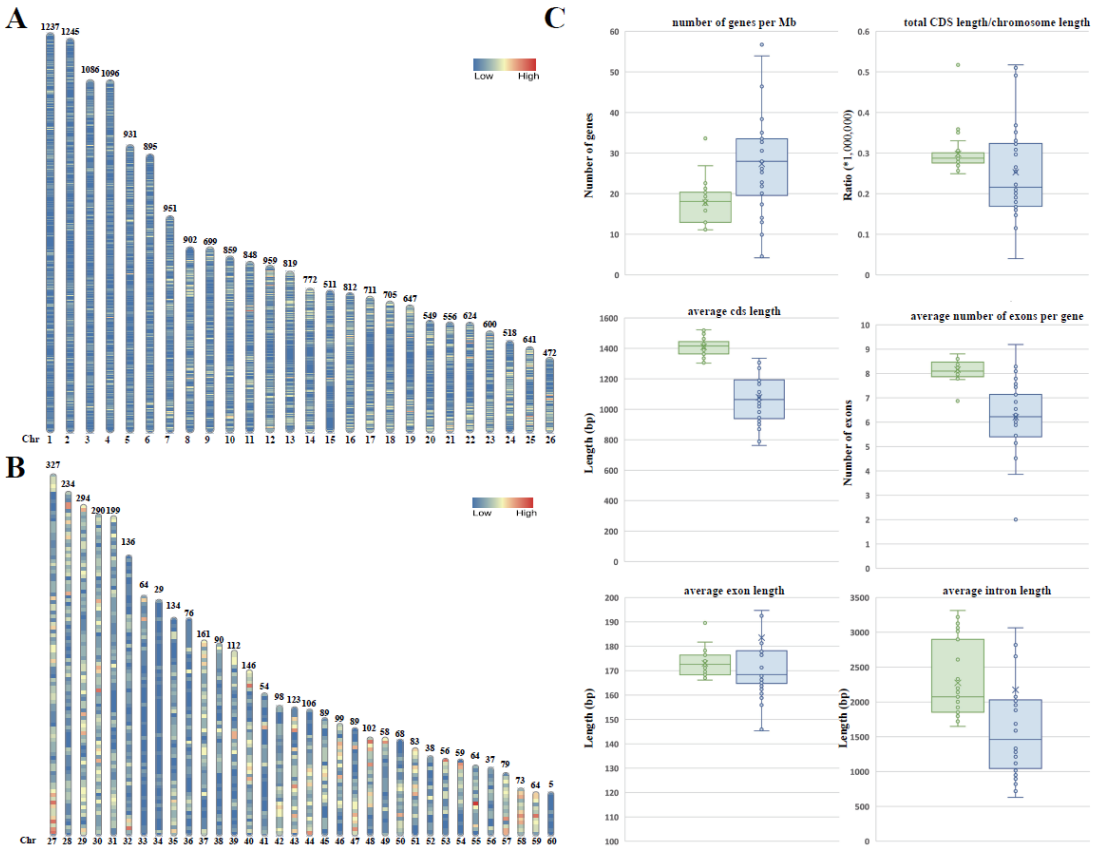

Figure S3. Gene density varies between macro- and micro-chromosomes of the American paddlefish. (A) Gene density of macrochromosomes. **(B)** Gene density of microchromosomes. The number on top of each chromosome refers to the number of genes on this chromosome. **(C)** Box-plots comparing gene number/Mb, total gene length/chromosome length, average CDS length, average exon number/gene, average exon length, and average intron length between macrochromosomes (green boxes) and microchromosomes (blue boxes).

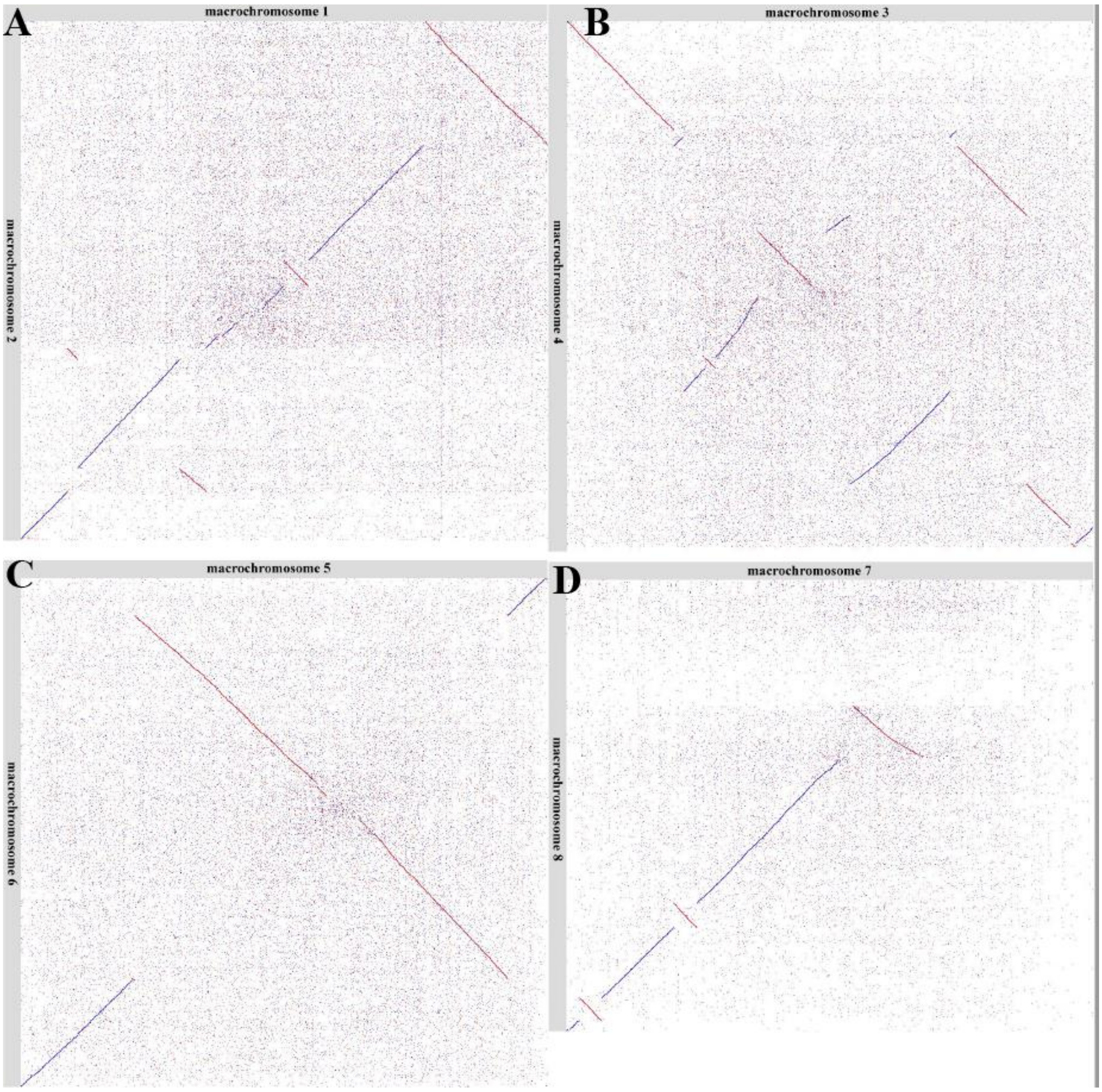

Figure S4. Dotplots showing sequence alignments between the four longest pairs of paralogous macrochromosomes. (A) Chr1 and Chr2; **(B)** Chr3 and Chr4; **(C)** Chr5 and Chr6; **(D)** Chr7 and Chr8. Forward matches are plotted in red, while reverse alignments are plotted in blue.

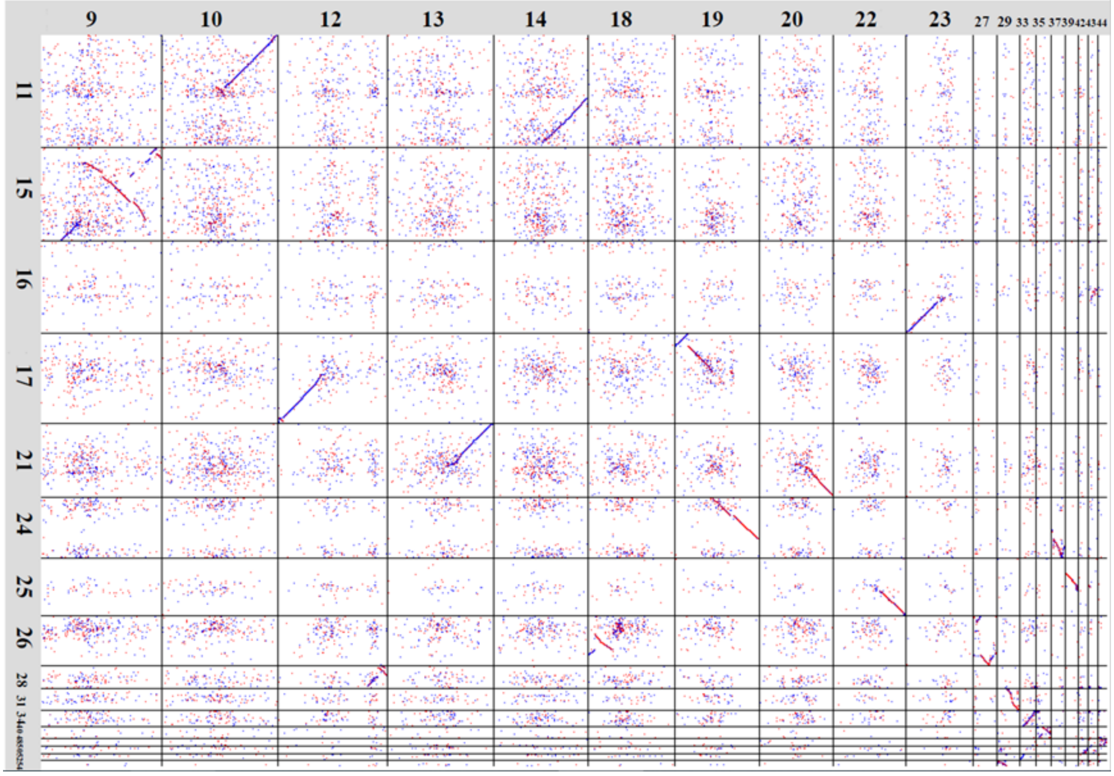

Figure S5. Dotplots showing sequence alignments between other chromosomes. X-axis: Chr9, 10, 12, 13, 14, 18, 19, 20, 22, 23, 27, 29, 33, 35, 37, 39, 42, 43, and 44; Y-axis: Chr11, 15, 16, 17, 21, 24, 25, 26, 28, 31, 34, 40, 48, 50, 52, and 54. Forward matches are plotted in red, while reverse alignments are plotted in blue.

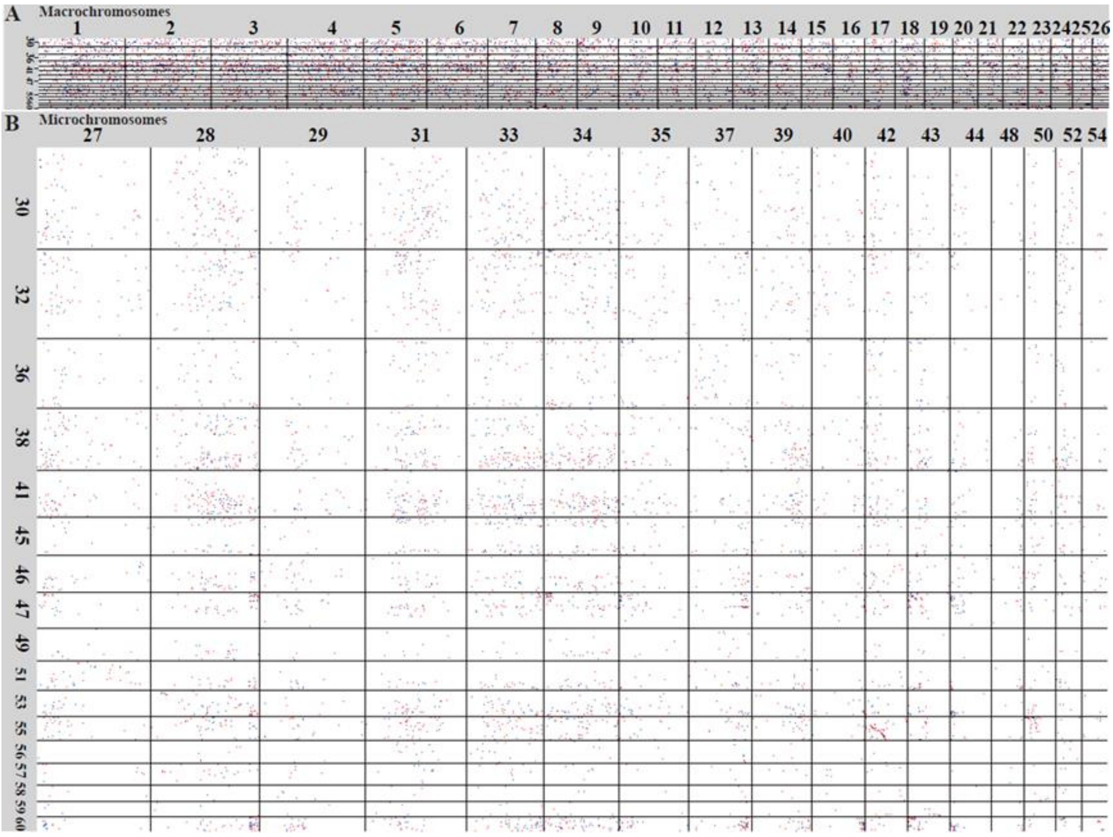

Figure S6. Dotplots showing unaligned sequences against all macrochromosomes (A) and microchromosomes (B). From top to bottom, unaligned chromosomes are Chr30, 32, 36, 38, 41, 45, 46, 47, 49, 51, 53, and 55–60.

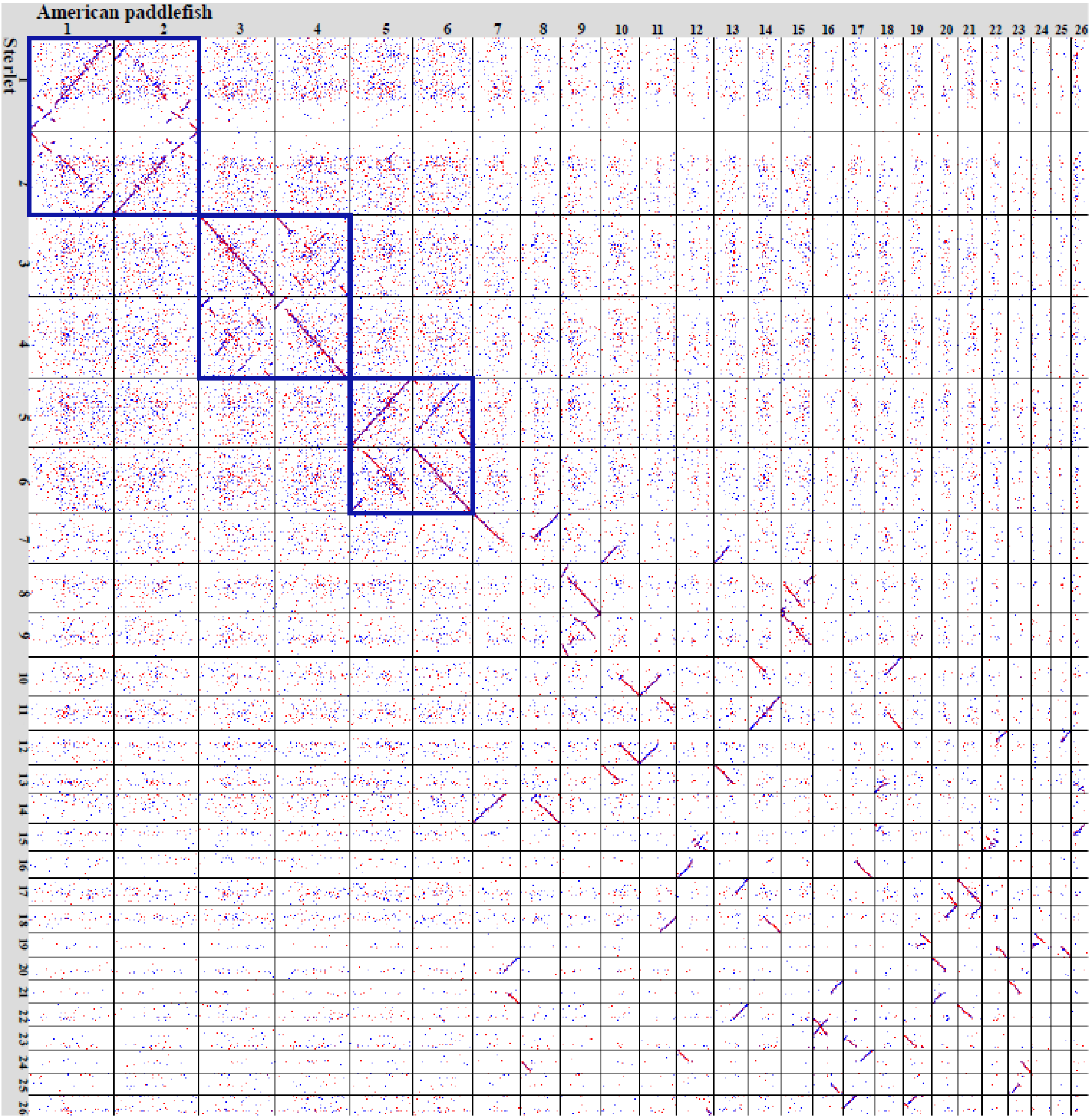

Figure S7. Dotplots showing sequence alignments between the 26 pairs of macrochromosomes of the American paddlefish and the sterlet. Boxes indicate the alignments of Chr1 and Chr2, Chr3 and Chr4, Chr5 and Chr6. Forward matches are plotted in red, while reverse alignments are plotted in blue.

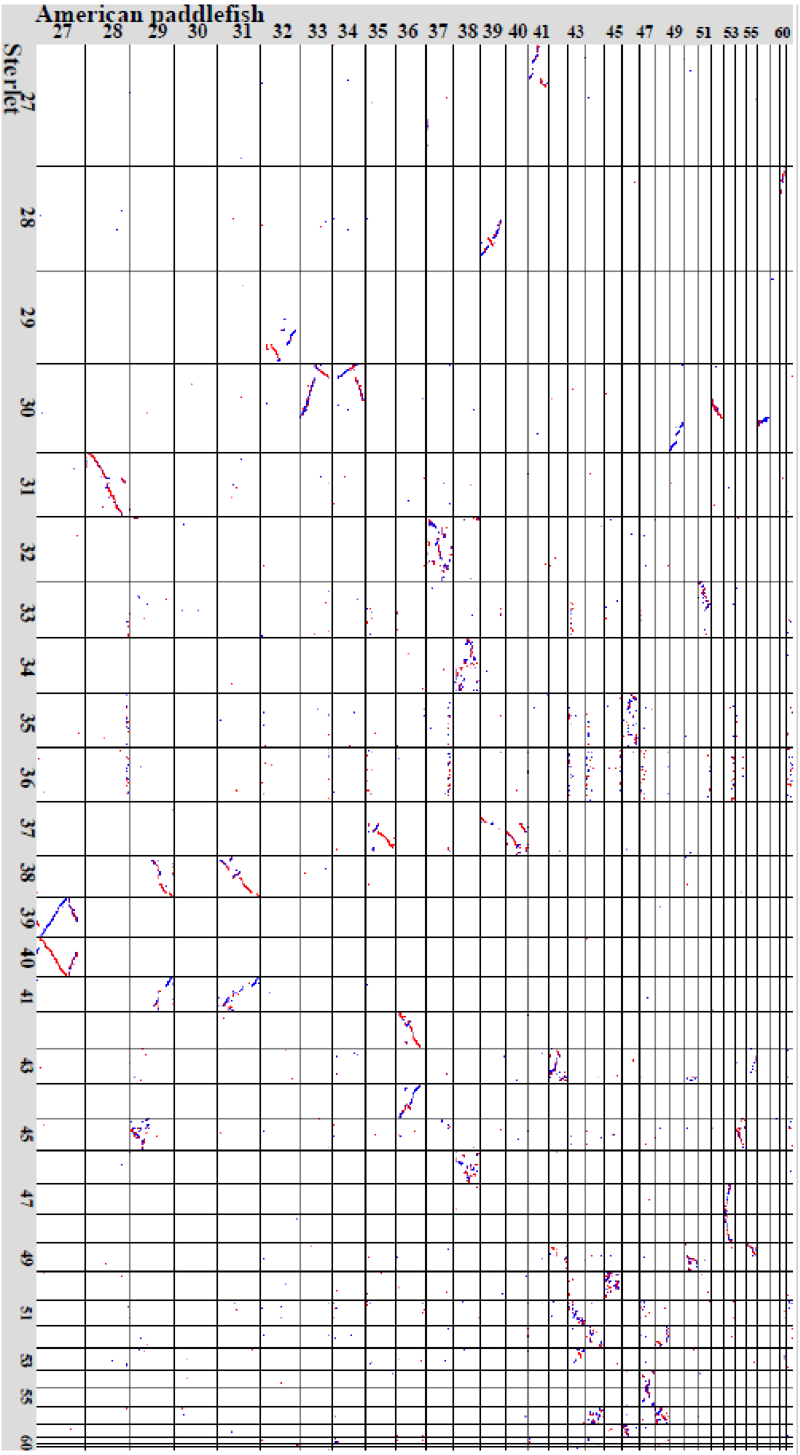

Figure S8. Dotplots showing sequence alignments between the 34 pairs of microchromosomes of the American paddlefish and the sterlet. Forward matches are plotted in red, while reverse alignments are plotted in blue.

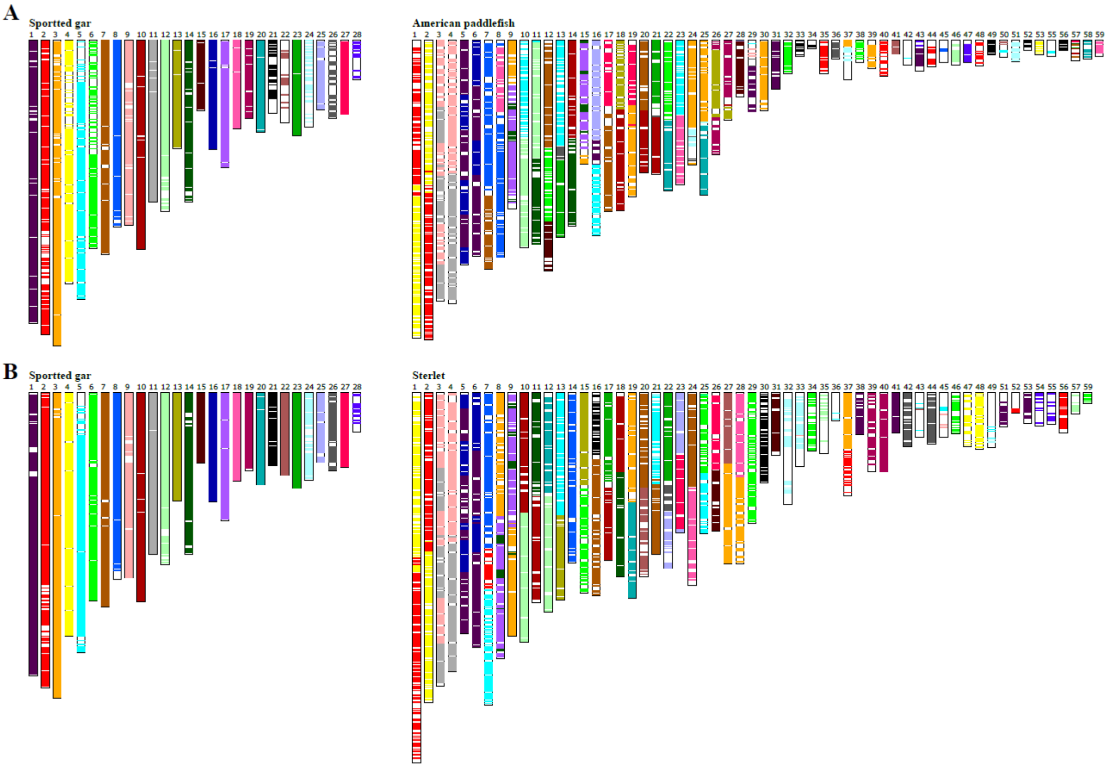

Figure S9. Synteny between chromosomes of (A) spotted gar and American paddlefish, and (B) spotted gar and sterlet. Each colored bar refers to a chromosome. The number on top of each bar indicates the chromosome number. Please note that chromosomal length is not the real length, since SynChro uses the number of annotated genes on each chromosome to interpret the synteny blocks. Therefore, if a chromosome (such as sterlet Chr7) is short in length but rich in genes, it will be longer than larger chromosomes with fewer genes (such as Chr5 and 6).

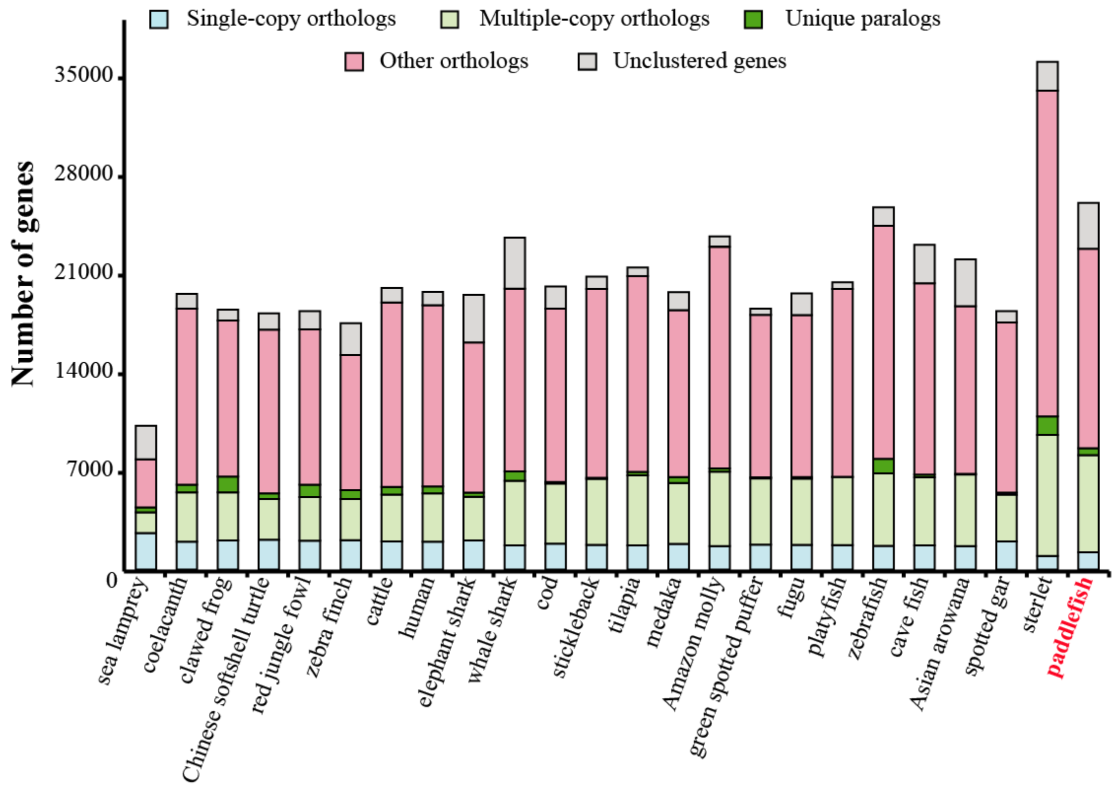

Figure S10. The number of genes clustered as orthologs and paralogs among paddlefish and other vertebrates.

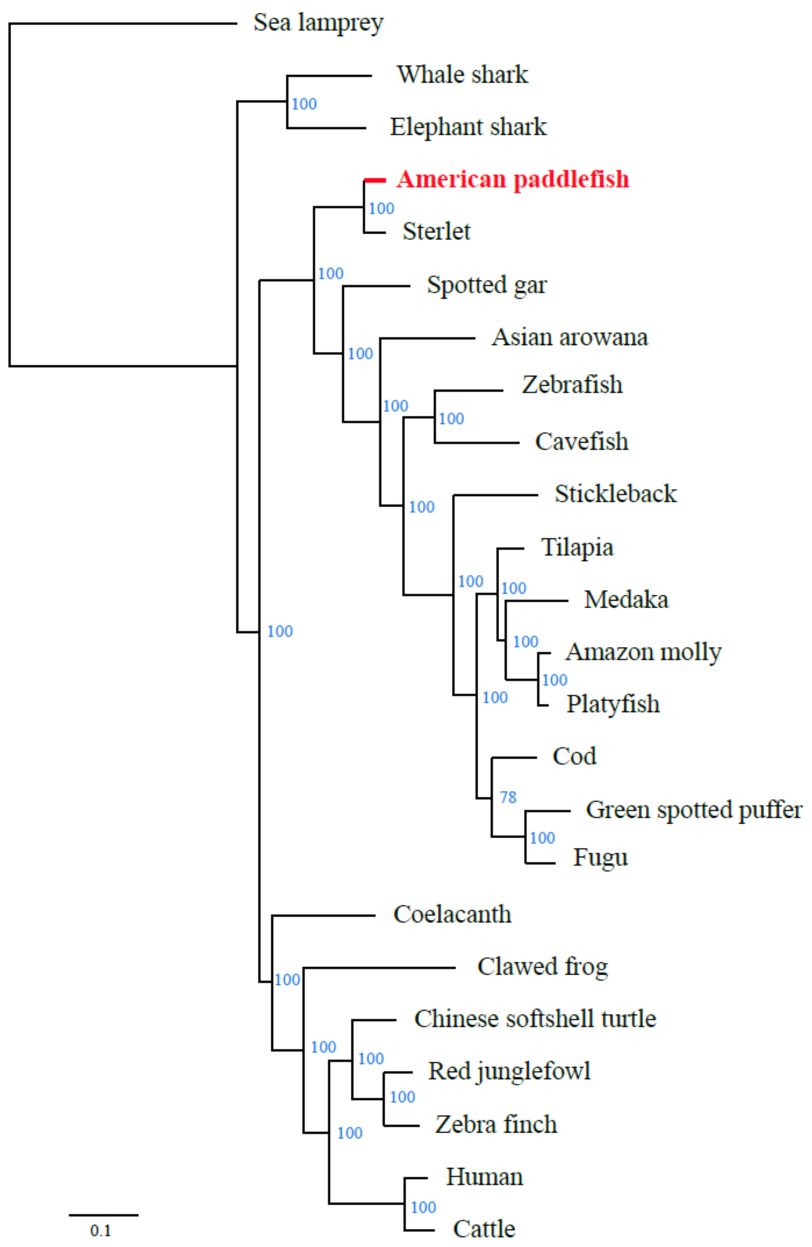

Figure S11. Original phylogenetic tree based on the maximum likelihood method. The red branch marks the position of the American paddlefish. Bootstrap supports are labeled at the nodes.

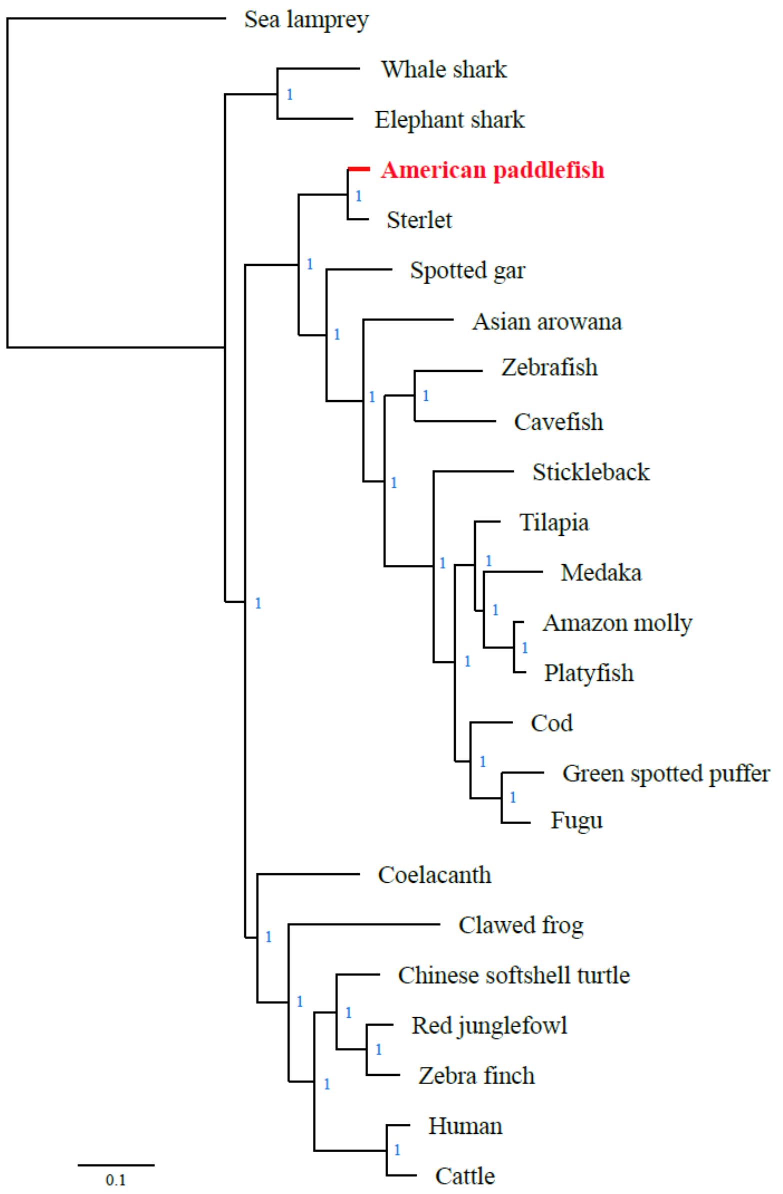

Figure S12. Original phylogenetic tree based on Bayesian inference. The red branch marks the position of the American paddlefish. Node support values are Bayesian posterior probabilities.

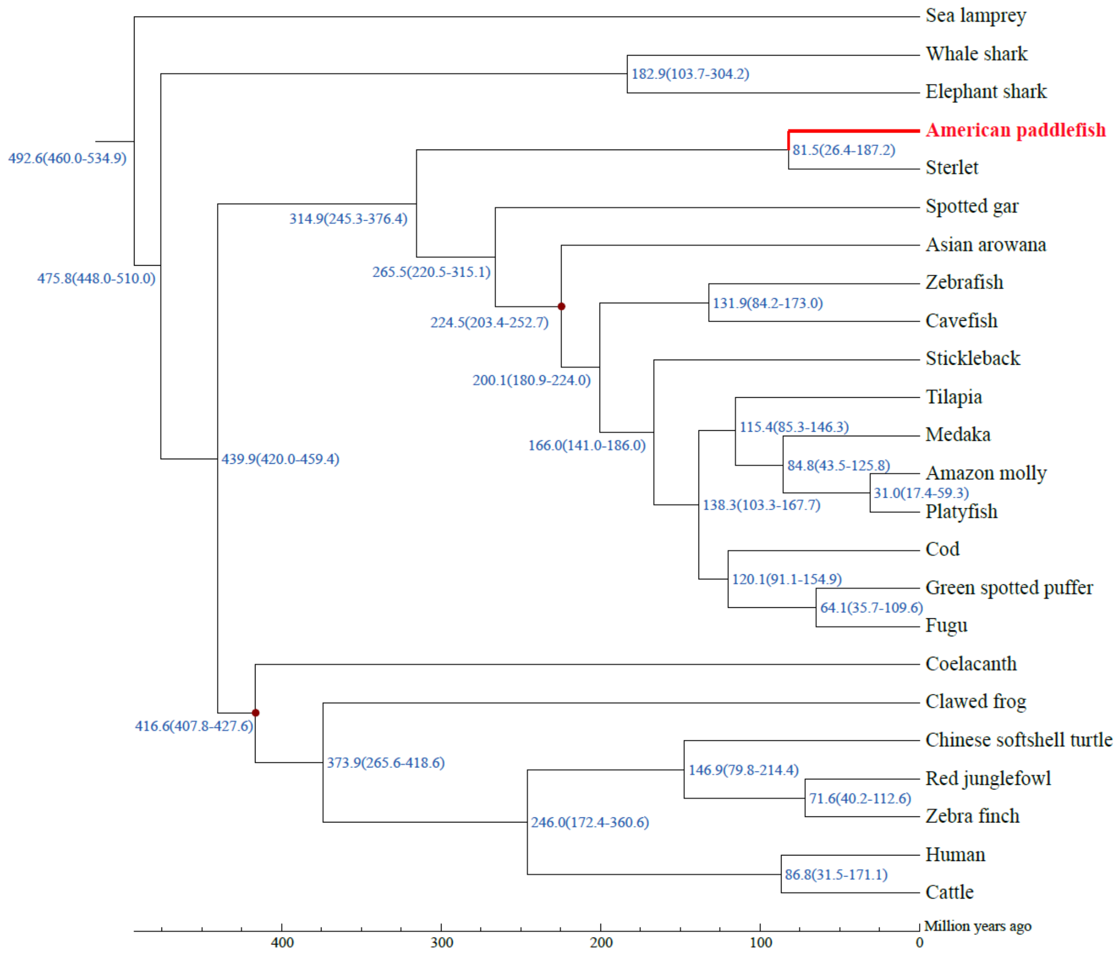

Figure S13. Divergence time estimation of the American paddlefish and other vertebrates. The red branch marks the position of the paddlefish. Numbers at each node indicate the divergence time followed by 95% confidence intervals in the bracket.

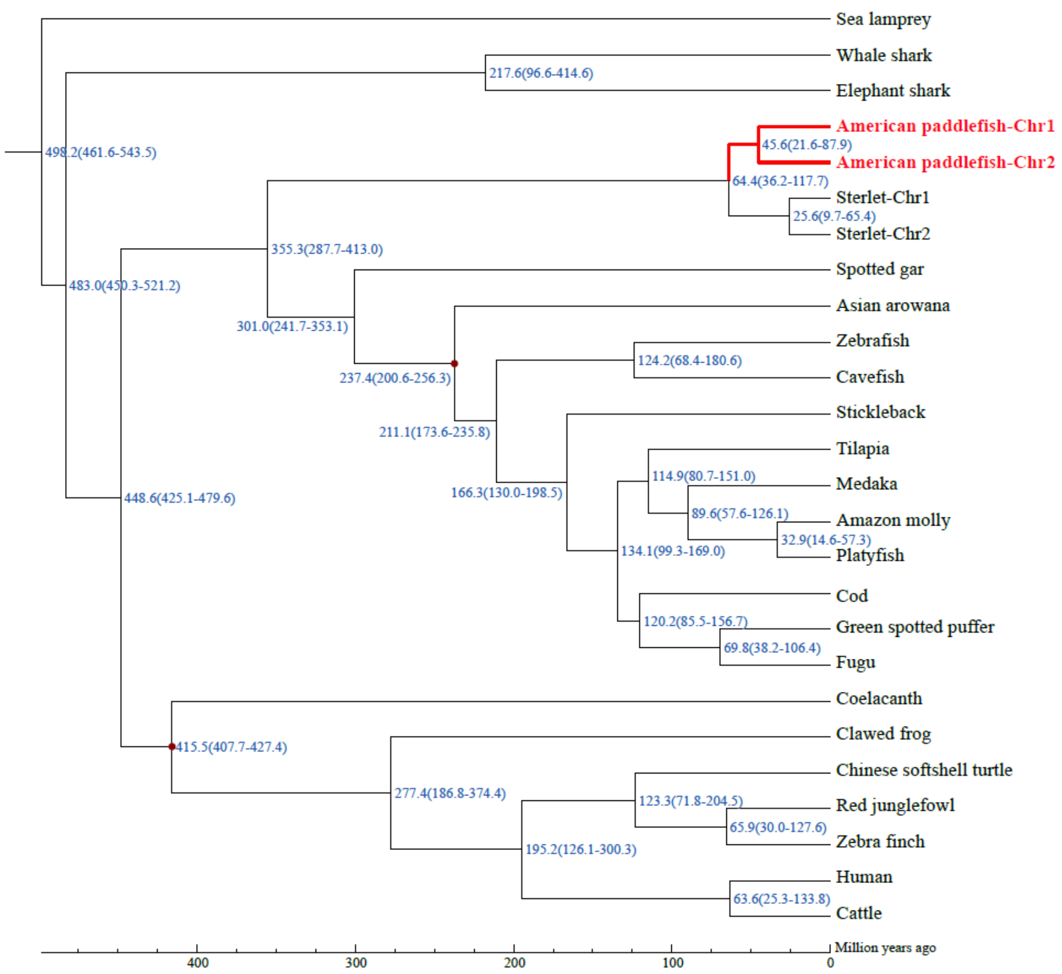

Figure S14. Divergence time estimation of the American paddlefish macrochromosomes 1 and 2. The red branches mark the position of paddlefish Chr1 and Chr2. Numbers at each node indicate the divergence time followed by 95% confidence intervals in the bracket.

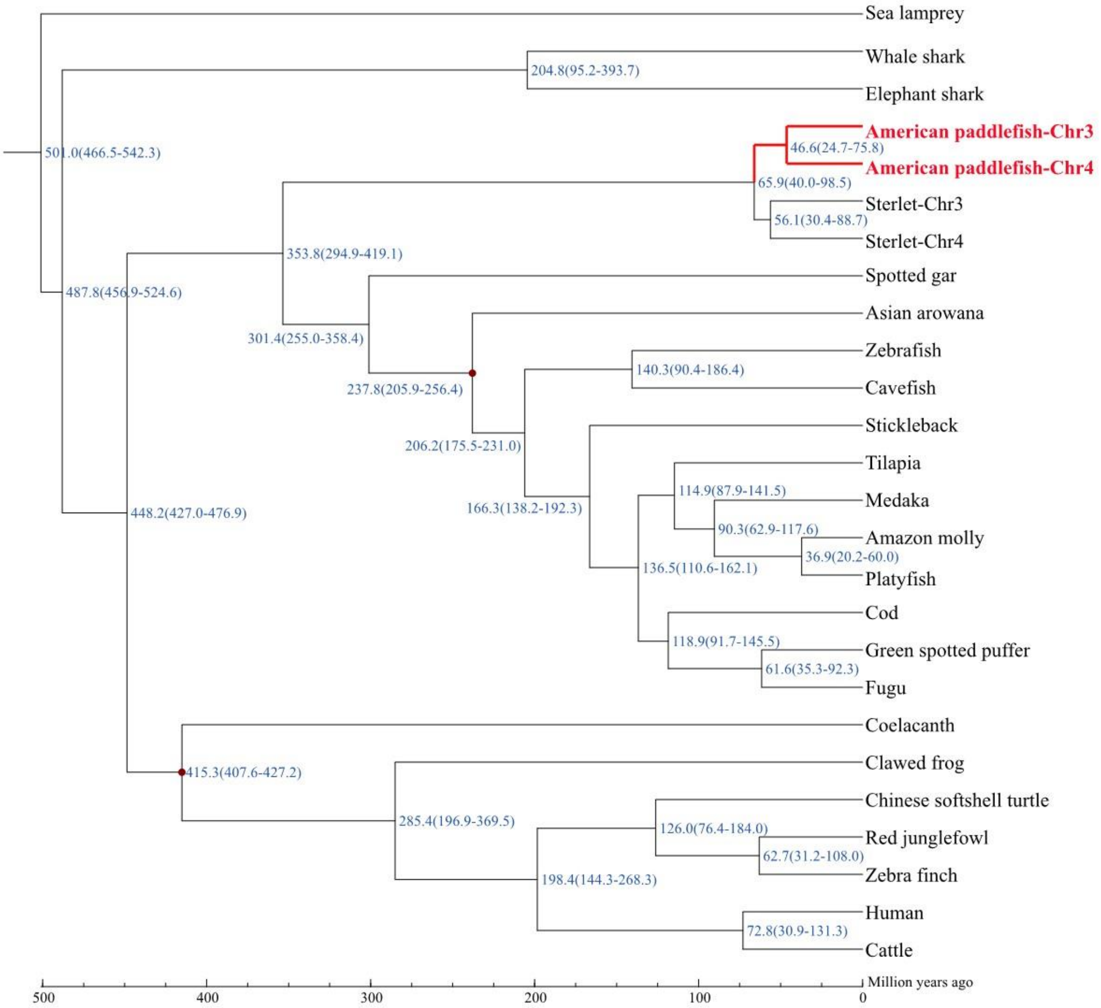

Figure S15. Divergence time estimation of the American paddlefish macrochromosomes 3 and 4. The red branches mark the position of paddlefish Chr3 and Chr4. Numbers at each node indicate the divergence time followed by 95% confidence intervals in the bracket.

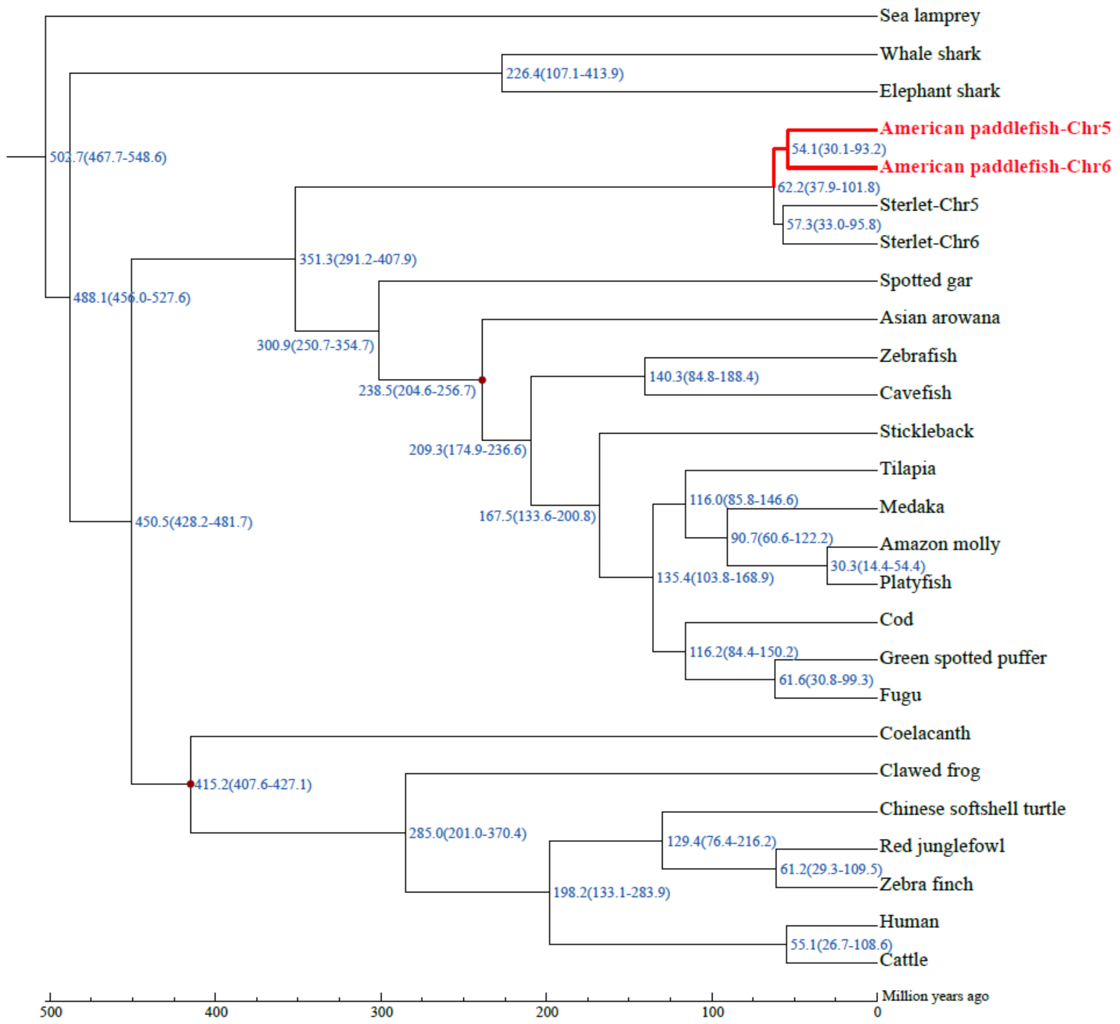

Figure S16. Divergence time estimation of the American paddlefish macrochromosomes 5 and 6. The red branches mark the position of paddlefish Chr5 and Chr6. Numbers at each node indicate the divergence time followed by 95% confidence intervals in the bracket.

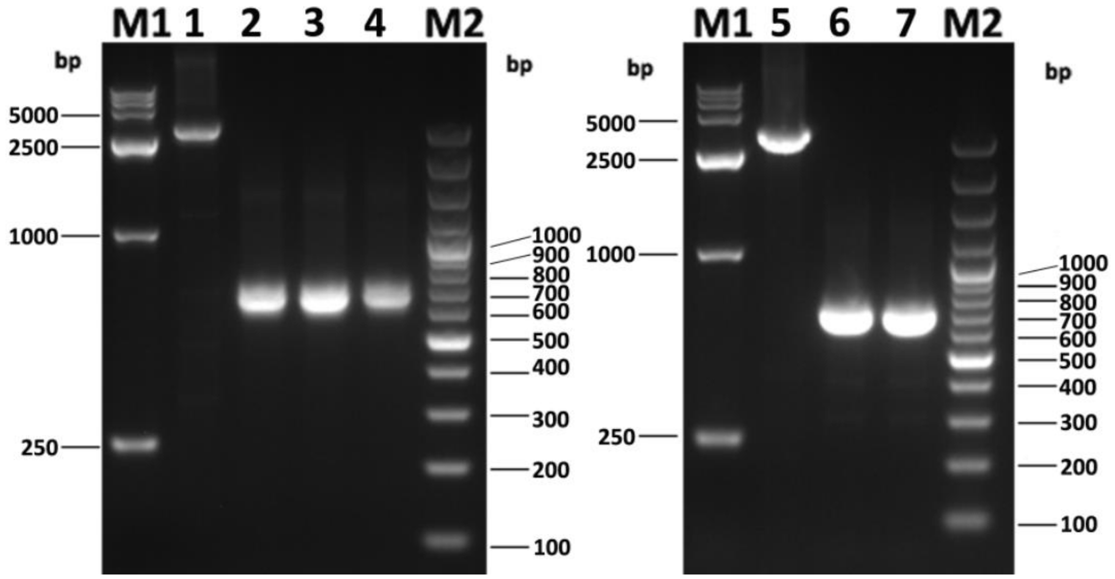

Figure S17. PCR amplification of the two *spp1* genes identified in the assembled genome. M1: marker D15000; M2: marker 100 bp plus; 1: spp1-1_1 (Chr1-nested-2 primers); 2: spp1-1_2 (PCR Chr1-F/R1 primers); 3: spp1-1_3 (PCR Chr1-F/R2 primers); 4: spp1-1_4 (PCR Chr1-F/R3 primers); 5: spp1-2_1 (Chr2-nested-2 primers); 6: spp1-2_2 (PCR Chr2-F/R1 primers); 7: spp1-2_3 (PCR Chr1-F/R2 primers).

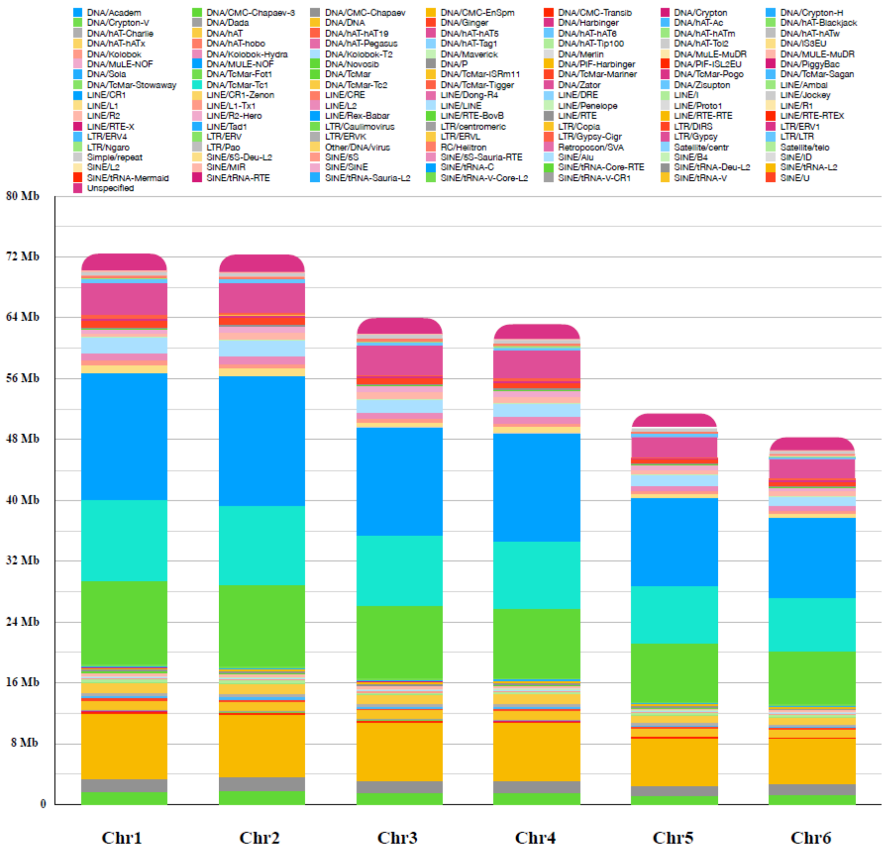

Figure S18. Repeat contents of the three largest pairs of paralogous chromosomes. Different colors in each bar refers to different supertypes of repeats in each chromosome. The Y-axis scales the total length of each supertype of the repeats.

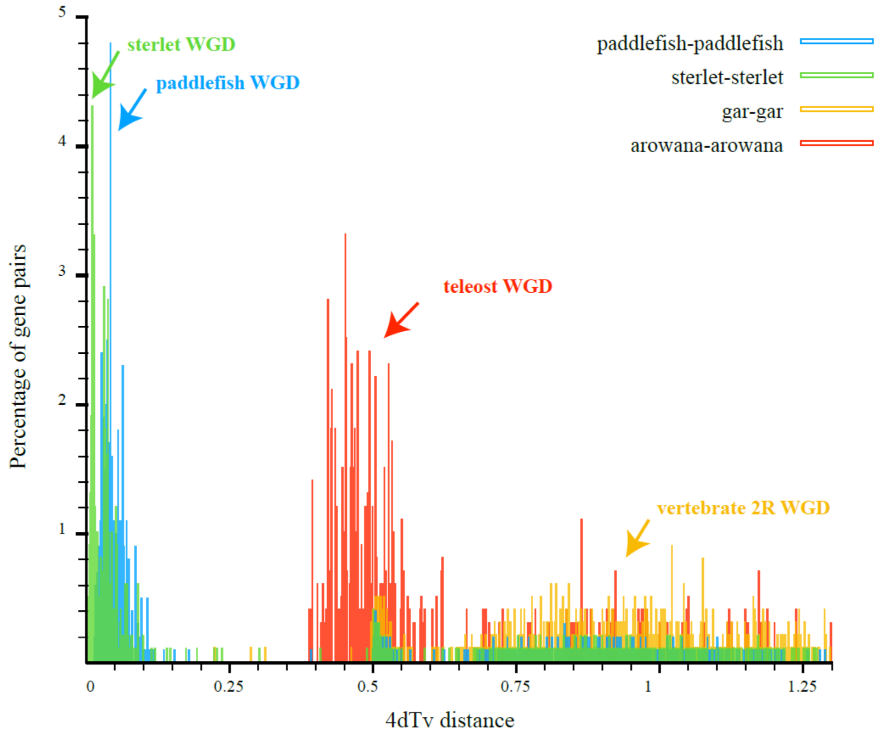

Figure S19. A 4dTv comparison between paddlefish, sterlet, spotted gar and Asian arowana. The X-axis denotes 4dTv distance corrected for multiple substitutions. The Y-axis indicates the percentage of gene pairs.

Table S1. Sequencing data generated from multiple sequencing platforms and various libraries for the paddlefish genome assembly.

**Sequencing libraries**	**Insert size**	**Raw data**	**Clean data**	**Mean read length**	**Sequence Coverage (X)**
Illumina Hiseq	270 bp	119.3	109.7	150 bp	77.5
	500 bp	31.5	28.7	125 bp	20.4
	800 bp	20.2	18.5	125 bp	13.1
	2 kb	37.8	23.3	150 bp	24.5
	5 kb	29.3	18.6	150 bp	19.0
	10 kb	34.5	19.8	150 bp	22.4
	20 kb	36.1	23.3	150 bp	23.4
PacBio reads	30 kb	54.3	52.1	10 kb	35.3
Hi-C reads	400 bp	99.3	65.3	100 bp	64.5
Total	—	462.3	359.3	—	300.2

Table S2. Statistics of the assembled American paddlefish genome.

Primary genome assembly	Scaffold		Contig	
	**Size (bp)**	**Number**	**Size (bp)**	**Number**
N90	1,107,274	329	915,285	393
N80	2,233,615	231	17,495,24	272
N70	3,181,010	173	2,736,516	202
N60	4,003,436	130	3,634,998	154
N50	4,861,270	95	4,296,843	114
Longest	22,462,060		17,819,526	
Total Size	1,540,770,834		1,540,393,193	
Number (>100 bp)	1,540,770,834	1,176	1,540,391,744	1,444
Number (> 2,000 bp)	1,540,770,834	1,143	1,540,391,744	1,444
**Chromosome-level genome assembly**	**Scaffold**		**Contig**	
	**Size (bp)**	**Number**	**Size (bp)**	**Number**
N90	7,546,224	32	255,888	706
N80	30,616,140	22	870,143	395
N70	36,461,845	18	1,591,728	264
N60	40,010,000	14	2,595,000	187
N50	48,906,729	10	3,441,286	136
Longest	110,695,931		13,167,000	
Total Size	1,542,128,830		1,540,391,744	
Number (>100 bp)	1,542,128,830	4,253	1,540,391,744	7,216
Number (> 2,000 bp)	1,541,109,645	3,365	1,539,159,655	6,111

Table S3. Completeness estimation of the assembled paddlefish genome with BUSCOs.

Category	BUSCOs hits	% of Genome
Complete BUSCOs (C)	4,295	93.7%
Complete and single-copy BUSCOs (S)	2,333	50.9%
Complete and duplicated BUSCOs (D)	1,962	42.8%
Fragmented BUSCOs (F)	106	2.3%
Missing BUSCOs (M)	183	4.0%
Total BUSCO groups searched	4,584	100%

Table S4. Detailed classification of repeat sequences in the American paddlefish genome assembly.

Type	Repeat size (bp)	% of Genome
ProteinMask	85,284,950	5.5303
RepeatMasker	181,235,849	11.7523
Tandem Repeat Finder	97,665,649	6.3332
De novo	536,533,739	34.7917
Total	592,405,540	38.4148

Table S5. Classification of predicted transposable elements in the American paddlefish genome. Repabse TEs represent RepeatMask against Repbase; Protein TEs represent RepeatProteinMask result against Repbase protein; *De novo* TEs represent RepeatMask against the *de novo* library; Combined TEs are the combined results from these three steps.

Type	Repbase TEs Length (bp)	Protein TEs Length (bp)	*De novo* TEs Length (bp)	Combined TEs Length (bp)	Percent (of genome)
DNA	107,598,104	8,941,296	211,415,181	265,537,807	17.2198%
LINE	56,290,337	54,214,152	191,943,903	223,771,095	14.5105%
SINE	1,903,956	0	2,823,997	4,603,174	0.2985%
LTR	26,525,675	22,164,366	123,075,347	140,247,770	9.0944%
Other	103,498	0	1,887	105,319	0.0068%
Total	181,235,849	85,284,950	516,612,604	541,382,610	35.1062%

Table S6. Statistics of the predicted gene set in the paddlefish genome.

Gene number	26,017
Average gene length	16,546 bp
Average coding sequence length	1,351 bp
Average exon number	7.83
Average exon length	172.63 bp
Average intron length	2,226.57 bp

Table S7. Statistics of gene functional annotations by various databases.

Values	Total	Swissprot-Annotated	KEGG-Annotated	TrEMBL-Annotated	Interpro-Annotated	Overall
Number	26,017	25,491	23,593	25,848	23,683	25,886
Percentage	100%	97.98%	90.68%	99.35%	91.03%	99.50%

Table S8. Statistics of distinguishable macro- and micro-chromosomes from the Hi-C assembly.

Chr ID	Length (bp)	of genome (%)	GC (%)	Gene number	Repeats (%)				
					SINE	LTR	LINE	DNA	All
scaffolds_1	110672920	7.1781	38.17	1237	0.14	10.38	17.14	19.13	40.61
scaffolds_2	109120529	7.0775	38.13	1245	0.22	10.15	17.89	18.92	40.58
scaffolds_3	97700507	6.3373	38.15	1086	0.15	10.47	16.67	19.26	40.36
scaffolds_4	97665480	6.3344	38.07	1096	0.15	10.31	16.81	18.92	40.13
scaffolds_5	79719676	5.1706	38.08	931	0.15	9.61	16.65	19.08	39.81
scaffolds_6	77117782	5.0015	38.02	895	0.15	9.64	15.92	18.66	38.65
scaffolds_7	60046701	3.8948	38.68	951	0.16	9.09	13.91	17.27	35.46
scaffolds_8	51457065	3.3386	38.66	902	0.29	9.02	12.54	17.73	35.27
scaffolds_9	51414706	3.3375	37.88	699	0.18	9.37	14.74	17.79	36.98
scaffolds_10	48894390	3.1714	38.32	859	0.15	8.56	12.79	16.73	33.48
scaffolds_11	47447217	3.0776	38.20	848	0.14	8.30	12.03	17.65	33.20
scaffolds_12	46382132	3.0085	38.91	959	0.23	7.53	11.04	16.27	31.03
scaffolds_13	44682680	2.8981	38.55	819	0.21	8.51	12.54	15.95	32.32
scaffolds_14	39999976	2.5945	38.53	772	0.14	8.57	12.93	16.42	33.23
scaffolds_15	39452593	2.5588	37.98	511	0.13	9.83	15.59	17.80	37.84
scaffolds_16	38779357	2.5159	39.81	812	0.45	5.73	9.72	13.21	26.80
scaffolds_17	37776802	2.4505	38.84	711	0.19	8.15	12.15	15.86	32.10
scaffolds_18	36445344	2.3644	38.97	705	0.25	8.15	12.30	15.77	32.22
scaffolds_19	35369582	2.2943	38.95	647	0.14	7.60	12.50	16.22	32.10
scaffolds_20	31159239	2.0215	39.23	549	0.23	7.74	11.91	14.69	30.35
scaffolds_21	30701252	1.9916	39.00	556	0.19	8.20	13.52	14.40	31.78
scaffolds_22	30597109	1.9853	39.41	624	0.19	8.03	13.40	15.21	32.31
scaffolds_23	28201217	1.8299	39.36	600	0.17	6.83	9.59	12.77	26.79
scaffolds_24	25485446	1.6540	40.11	518	0.31	8.06	14.03	15.83	34.21
scaffolds_25	23799018	1.5453	41.46	641	0.26	6.96	9.76	12.10	26.53
scaffolds_26	20874733	1.3556	40.00	472	0.26	6.99	14.24	14.87	31.99
scaffolds_27	9652038	0.6309	42.38	327	0.39	5.09	8.28	12.16	24.19
scaffolds_28	9241349	0.6005	40.09	234	0.34	7.37	12.66	13.88	31.64
scaffolds_29	8882762	0.5774	43.41	294	1.25	8.38	15.16	20.65	45.03
scaffolds_30	8557350	0.5609	39.38	290	0.27	4.61	5.22	10.50	19.26
scaffolds_31	8583030	0.5579	41.99	199	0.92	8.90	16.97	16.82	40.24
scaffolds_32	7490990	0.4893	40.92	136	0.29	7.08	9.19	12.26	27.83
scaffolds_33	6458086	0.4192	39.07	64	0.19	7.30	19.69	15.00	37.83
scaffolds_34	6343732	0.4121	40.14	29	0.56	11.55	26.62	16.54	49.01
scaffolds_35	5847329	0.3808	41.41	134	1.10	8.88	15.64	13.15	36.54
scaffolds_36	5810723	0.3797	42.25	76	0.24	6.14	8.76	13.56	28.44

scaffolds_37	5236841	0.3413	43.11	161	1.21	8.62	12.74	15.66	37.05
scaffolds_38	5123632	0.3360	42.83	90	0.91	9.28	19.03	17.59	44.50
scaffolds_39	4985363	0.3234	41.17	112	0.38	7.72	13.21	13.57	31.91
scaffolds_40	4455632	0.2893	42.04	146	1.54	5.37	10.61	16.09	31.36
scaffolds_41	3805706	0.2492	40.18	54	0.32	6.11	13.50	11.89	29.32
scaffolds_42	3499438	0.2274	42.42	98	1.97	11.17	19.40	20.43	52.49
scaffolds_43	3433677	0.2250	43.65	123	2.37	12.35	18.31	24.22	54.78
scaffolds_44	3390696	0.2210	44.61	106	2.20	8.05	20.16	22.07	52.91
scaffolds_45	3150032	0.2060	44.58	89	1.67	16.34	17.71	24.68	61.95
scaffolds_46	2980232	0.1961	44.56	99	0.82	11.07	15.62	30.90	60.15
scaffolds_47	2846283	0.1887	44.12	89	1.02	11.05	19.31	18.47	50.37
scaffolds_48	2650282	0.1723	44.57	102	2.81	8.88	17.79	21.16	49.95
scaffolds_49	2640359	0.1722	41.52	58	0.22	3.38	3.24	10.28	16.25
scaffolds_50	2595998	0.1687	43.27	68	1.09	12.40	27.27	23.58	61.92
scaffolds_51	2344646	0.1536	45.90	83	1.69	10.19	14.78	22.69	47.54
scaffolds_52	2152814	0.1396	39.14	38	0.20	7.11	25.19	8.17	36.72
scaffolds_53	2057129	0.1353	42.49	56	1.48	9.28	16.71	14.91	39.27
scaffolds_54	2027415	0.1341	44.63	59	0.95	15.82	22.39	22.66	64.54
scaffolds_55	1901070	0.1237	42.64	64	0.24	11.84	21.60	19.75	49.39
scaffolds_56	1836670	0.1195	38.04	37	0.21	2.68	4.50	8.91	15.91
scaffolds_57	1669624	0.1104	42.17	79	0.23	4.30	5.48	9.55	18.00
scaffolds_58	1264954	0.0835	43.19	73	0.32	2.43	3.25	5.24	10.72
scaffolds_59	1165121	0.0769	43.30	64	1.15	5.24	4.34	9.84	18.47
scaffolds_60	1176238	0.0767	41.20	5	0.14	12.99	42.50	9.05	58.22
unanchored	54172499	3.5339	42.69	1636	1.58	13.32	15.46	17.20	45.96

Table S9. Statistics of gene sets clustering among paddlefish and other vertebrates.

Species	Total genes	Clustered genes	Unclustered genes	Family No.	Unique families	Average genes/family
Astyanax mexicanus	23041	20305	2736	14248	77	1.43
Danio rerio	25709	24391	1318	14691	181	1.66
Gadus morhua	20083	18515	1568	13538	41	1.37
Gasterosteus aculeatus	20785	19908	877	13982	28	1.42
Latimeria chalumnae	19568	18516	1052	13307	136	1.39
Lepisosteus oculatus	18341	17532	809	13817	53	1.27
Oreochromis niloticus	21437	20816	621	13776	68	1.51
Oryzias latipes	19682	18404	1278	13079	110	1.41
Pelodiscus sinensis	18177	17017	1160	12420	78	1.37
Poecilia formosa	23613	22894	719	15381	78	1.49
Taeniopygia guttata	17472	15226	2246	11515	68	1.32
Takifugu rubripes	18518	18065	453	12724	28	1.42
Tetraodon nigroviridis	19595	18051	1544	12827	46	1.41
Xenopus tropicalis	18442	17667	775	12026	143	1.47
Xiphophorus maculatus	20379	19917	462	14601	8	1.36
Bos taurus	19981	18954	1027	14357	140	1.32
Callorhinchus milii	19491	16111	3380	12717	102	1.27
Rhincodon typus	23554	19922	3632	13352	196	1.49
Gallus gallus	18342	17042	1300	12384	143	1.38
Homo sapiens	19696	18762	934	14446	128	1.3
Petromyzon marinus	10202	7812	2390	6354	98	1.23
Scleropages formosus	22016	18680	3336	12939	26	1.44
Acipenser ruthenus	36020	33974	2046	15053	476	2.26
Polyodon spathula	26017	22772	3245	12314	240	1.85

Table S10. Information of *spp1* and nearby genes in the American paddlefish and the spotted gar.

Gene	American paddlefish Chr1			American paddlefish Chr2			Spotted gar LG2
	Loci-start	Loci-end	Strand	Loci-start	Loci-end	Strand	Ensembl Accession
pde6b	34,284,708	34,300,583	+	94,947,096	94,962,291	+	ENSLOCG00000012238.1
paip1	37,748,735	37,761,145	+	97,855,103	97,868,316	+	ENSLOCG00000012270.1
nnt	37,668,439	37,739,278	-	97,804,118	97,845,883	-	ENSLOCG00000012282.1
fgf10b	37,526,734	37,545,640	+	97,677,570	97,696,560	+	ENSLOCG00000012296.1
rai14	12,577,675	12,602,135	+	93,268,768	93,299,105	-	ENSLOCG00000012300.1
amacr	12,480,133	12,481,995	-	93,434,198	93,457,638	+	ENSLOCG00000012318.1
slc45a2	-	-	/	93,466,230	93,477,800	+	ENSLOCG00000012323.1
rxfp3	12,453,547	12,454,770	+	93,488,971	93,490,194	-	ENSLOCG00000017549.1
adamts12	12,371,075	12,443,824	-	93,500,796	93,657,994	+	ENSLOCG00000012331.1
gzmk	12,304,636	12,306,825	-	93,700,692	93,703,481	+	ENSLOCG00000012345.1
tmem267	12,607,896	12,609,002	+	93,262,275	93,263,370	-	ENSLOCG00000012351.1
il11ra	12,625,999	12,647,803	-	93,230,840	93,249,510	+	ENSLOCG00000012354.1
cntfr	12,749,080	12,815,479	-	93,061,830	93,130,334	+	ENSLOCG00000012362.1
galt	13,044,212	13,092,130	-	92,731,420	92,795,164	+	ENSLOCG00000012369.1
sigmar1	13,095,637	13,098,663	+	92,724,416	92,728,361	-	ENSLOCG00000012381.1
katnal2	13,103,619	13,110,073	+	92,708,248	92,720,999	-	ENSLOCG00000012385.1
hdhd2	13,117,348	13,121,381	-	92,695,065	92,702,379	+	ENSLOCG00000012392.1
smad2	13,338,992	13,372,800	-	92,454,095	92,479,175	+	ENSLOCG00000012409.1
cldn23a	16,461,492	16,462,307	-	89,149,347	89,150,138	+	ENSLOCG00000017550.1
rchy1	53665995	53685398	+	57,502,034	57,524,301	-	ENSLOCG00000012485.1
spp1	53,659,617	53,663,837	+	57,527,701	57,531,323	-	ENSLOCG00000012495.1
sh3bp2	53,536,882	53,571,399	-	57,647,785	57,669,358	+	ENSLOCG00000012502.1
vldlr	16,420,627	16,442,388	+	60,663,138	60,776,072	+	ENSLOCG00000012505.1
kcnv2a	-	-	/	60,820,201	60,823,162	+	ENSLOCG00000012521.1
pum3	49,281,384	49,310,937	+	60,850,489	60,903,977	-	ENSLOCG00000012529.1
carm1l	-	-	/	-	-	/	ENSLOCG00000012543.1
fybb	38,922,582	38,949,320	-	98,892,074	98,917,662	-	ENSLOCG00000012577.1
rictorb	38,834,090	38,907,004	-	98,799,509	98,875,202	-	ENSLOCG00000012599.1
osmr	38,799,782	38,824,377	+	98,739,106	98,763,688	-	ENSLOCG00000012617.1
lifra	38,723,782	38,746,894	-	98,733,816	98,766,722	-	ENSLOCG00000012627.1
egflam	38,668,095	38,713,126	+	98,673,535	98,725,395	+	ENSLOCG00000012642.1
gdnfa	38,574,448	38,576,900	-	98,597,727	98,600,850	-	ENSLOCG00000012652.1
wdr70	38,435,361	38,549,239	+	98,444,378	98,571,409	+	ENSLOCG00000012656.1
nup155	38,403,642	38,432,304	-	98,412,648	98,439,874	-	ENSLOCG00000012677.1
cplane1	38,350,826	38,393,368	-	-	-	/	ENSLOCG00000012697.1
nipbla	38,284,525	38,346,098	+	98,332,173	98,397,179	+	ENSLOCG00000012704.1
slc1a3b	38,193,618	38,221,938	+	98,216,541	98,248,015	+	ENSLOCG00000012724.1
ranbp3l	-	-	/	98,132,763	98,136,729	-	ENSLOCG00000012736.1
nadk2	38,099,489	38,110,368	-	98,111,707	98,122,098	-	ENSLOCG00000012738.1
skp2	38,085,948	38,092,188	+	98,099,415	98,104,589	+	ENSLOCG00000012750.1
lmbrd2a	38,054,460	38,078,282	-	98,070,106	98,091,808	-	ENSLOCG00000012758.1

Table S11. Primers used for amplification of *spp1-1* and *spp1-2*.

Target Gene	Method	Primer	Sequence	Location (bp)	Length (bp)
spp1-1 4224bp	Chr1-nested-1	nested-F1	ATGAATACAGCAATTCTGTTATTCT	1..3285	3285
		nested-R1	TTGCTAGATTGTCACCCCTGTC		
	Chr1-nested-2	nested-F2	CCTTCCAGTGAGTATTTTACTTTGTT	48..3208	3161
		nested-R2	GTCGGTTGTGCCGTTGTG		
	PCR	Chr1-F1	ATGAATACAGCAATTCTGTTATTC	1..717	717
		Chr1-R1	TATTGATTGATGTATGACAAAGAC		
		Chr1-F2	ATAGTACCGTACAAATAGTGTATG	2994..3705	712
		Chr1-R2	ACATGGTGGCCCACTTATTCATGA		
		Chr1-F3	TATTAATGCATTCATGTTGACTGT	3511..4224	714
		Chr1-R3	TTACTTTTATTTCAGTGACTGAGC		
spp1-2 3962bp	Chr2-nested-1	nested-F1	ATGAATACAACAATTCTGATATTCT	1..3962	3962
		nested-R1	TTACTTTTGTTTCAGTGACTGAGCA		
	Chr2-nested-2	nested-F2	GAGGAACAGGTATGCAGGTTGT	197..3726	3530
		nested-R2	TGTCATCACTTTCTTTGGGAGC		
	PCR	Chr2-F1	ATGAATACAACAATTCTGATATTC	1..718	718
		Chr2-R1	CTTAAGTCCCAAAATAGTCAATGT		
		Chr2-F2	ATTAATGTATTAATTTTGACTCTGAT	3243..3962	720
		Chr2-R2	TTACTTTTGTTTCAGTGACTGAGC		
